# Enrichment and differential targeting of complexins 3 and 4 in ribbon-containing sensory neurons during zebrafish development

**DOI:** 10.1186/1749-8104-5-24

**Published:** 2010-09-01

**Authors:** George Zanazzi, Gary Matthews

**Affiliations:** 1 Graduate Program in Neuroscience, State University of New York, Stony Brook, NY 11794, USA; 2 Department of Neurobiology and Behavior, State University of New York, Stony Brook, NY 11794, USA

## Abstract

**Background:**

In sensory systems with broad bandwidths, polarized receptor cells utilize highly specialized organelles in their apical and basolateral compartments to transduce and ultimately transmit signals to the rest of the nervous system. While progress has been made in elucidating the assembly of the transduction apparatus, the development of synaptic ribbon-containing terminals remains poorly understood. To begin to delineate the targeting of the exocytotic machinery specifically in ribbon-containing neurons, we have examined the expression of complexins 3 and 4 in the zebrafish visual and acousticolateral systems during the first week of development.

**Results:**

We have identified five members of the complexin 3/4 subfamily in zebrafish that show 50 to 75% amino acid identity with mammalian complexins 3 and 4. Utilizing a polyclonal antibody that recognizes all five orthologs, we demonstrate that these proteins are enriched in ribbon-containing sensory neurons. Complexin 3/4 is rapidly targeted to presynaptic terminals in the pineal organ and retina concomitantly with RIBEYE b, a component of ribbons. In hair cells of the inner ear and lateral line, however, complexin 3/4 immunoreactivity clusters on the apical surfaces of hair cells, among their stereocilia, rather than along the basolateral plasma membrane with RIBEYE b. A complexin 4a-specific antibody selectively labels the presynaptic terminals of visual system ribbon-containing neurons.

**Conclusions:**

These results provide evidence for the concurrent transport and/or assembly of multiple components of the active zone in developing ribbon terminals. Members of the complexin 3/4 subfamily are enriched in these terminals in the visual system and in hair bundles of the acousticolateral system, suggesting that these proteins are differentially targeted and may have multiple roles in ribbon-containing sensory neurons.

## Background

Photosensitive and non-cutaneous mechanosensitive neurons transduce stimuli, via specialized sensory cilia and microvilli on their apical surfaces, into graded membrane potentials. A rod or cone photoreceptor elaborates, from a primary cilium, vast quantities of plasma membrane that contain opsins and the rest of the transduction machinery (reviewed in [1]). Less well-developed outer segments are also present in pineal complex photoreceptors in anamniotes and sauropsids (reviewed in [2]). Developing mechanosensory hair bundles, on the other hand, consist of a kinocilium and actin-rich stereocilia embedded in a cuticular plate and interconnected with proteinaceous linkages (reviewed in [3,4]). In teleosts such as zebrafish, mechanosensory hair cells are located in sensory patches of inner ear epithelia (maculae and cristae) and in neuromasts of the lateral lines (reviewed in [4]). Defects in the assembly of hair cell bundles and photoreceptor outer segments can lead to receptor cell degeneration and sensory deficits (reviewed in [1,3,4]).

The presynaptic terminals of receptor cells in the visual and acousticolateral systems have an architecture that allows them to effectively transmit information about stimulus intensity and frequency with release modification. Synaptic vesicle exocytosis occurs at active zones that contain ribbons. These pleiomorphic organelles, composed primarily of a unique protein called RIBEYE, tether a halo of releasable vesicles (reviewed in [5,6]). Teleost genomes contain two *RIBEYE *genes, *RIBEYE a *and *RIBEYE b*, that are differentially expressed in zebrafish [7]. The importance of the ribbon in synaptic transmission is underscored by the loss of vision [8,9] and hearing [10] in zebrafish and mouse mutants that lack anchored ribbons. Despite the importance of ribbons in visual, auditory, and vestibular function, the mechanisms that underlie their development, and the rest of the exocytotic machinery, are not well-understood.

Ultrastructural studies of ribbon development in photoreceptors [11,12], hair cells [13], and pinealocytes [14] suggest that ribbons (floating, spherical, and surrounded by vesicles) are found first in the perinuclear cytoplasm. As development proceeds, ribbons appear to migrate basally in photoreceptor axons toward the presynaptic terminals [12]. Ribbon cytomatrix proteins, such as RIBEYE, are found in migrating non-membranous densities, termed precursor spheres [15], which may correspond to migrating ribbons. In the presynaptic terminal, a ribbon attaches to the plasma membrane via an arciform density [16] and enlarges with maturation, likely due to multiple RIBEYE homophilic and heterophilic interactions [17]. Besides ribbons, several groups have examined the accumulation of synaptic vesicle proteins in the developing rodent retina, revealing clustering in the outer plexiform layer (OPL) and inner plexiform layer (IPL) before synapse maturation [18-21]. Since the examined synaptic vesicle proteins are found at both conventional and ribbon synapses, the targeting of ribbon terminal-specific synaptic vesicle proteins is poorly understood.

Although the molecular architectures of ribbon and conventional terminals are similar, specific isoforms of synaptic vesicle proteins have been identified at some ribbon synapses (reviewed in [5,6]). For example, adult mammalian retinal ribbon synapses selectively express the complexin 3/4 subfamily [22,23]. The mammalian complexins are a family of small, hydrophilic proteins encoded by four genes that can be grouped into two subfamilies based upon sequence identity and expression patterns [23-26]. Complexins 1 and 2 share approximately 80% amino acid identity and are enriched at conventional synapses [24-26], where they appear to activate and stabilize the SNARE complex in a fusion-ready state before calcium enters the terminal and binds to synaptotagmin (reviewed in [27]). Mammalian complexins 3 and 4 are homologous to each other (approximately 60% amino acid identity), but not to the complexin 1/2 subfamily (approximately 25% amino acid identity). Mammalian complexin 3 localizes primarily to rod bipolar cell and cone photoreceptor terminals, while mammalian complexin 4 is expressed in cone bipolar cell and rod photoreceptor terminals [23]. In confirmation of their importance in retinal ribbon synaptic transmission, mice with targeted disruptions of the complexin 3 and 4 genes exhibit vision deficits and disorganized photoreceptor terminals containing floating ribbons [28].

In this study, we characterize zebrafish complexin 3/4 orthologs and their expression in sensory ribbon-containing neurons during embryonic and early larval zebrafish development. By searching the ENSEMBL and GenBank databases, we identified and subsequently cloned five zebrafish orthologs that show 50 to 75% amino acid identity with mammalian complexins 3 and 4. Phylogenetic analysis reveals two complexin 3 orthologs and three complexin 4 orthologs. Utilizing a polyclonal antibody that recognizes all five zebrafish orthologs, we demonstrate that these proteins are rapidly and predominantly targeted to the lateral border of the pineal organ and to the OPL and IPL in the embryonic zebrafish retina. Complexin 3/4 overlaps with zpr 1/FRet 43 in terminals of double cone photoreceptors in the OPL and pineal, and with protein kinase C in ON bipolar cell terminals in the IPL. Hair cells of the acousticolateral system display complexin 3/4 immunoreactivity among their stereocilia rather than in their basolateral domains containing RIBEYE b. While complexin 4a marks visual system ribbon terminals, it is not found in hair cells, suggesting a system-specific subdivision of cellular functions.

## Results

### Cloning, phylogenetic, and syntenic analyses of zebrafish complexin 3 and 4 orthologs

**Figure 1:**
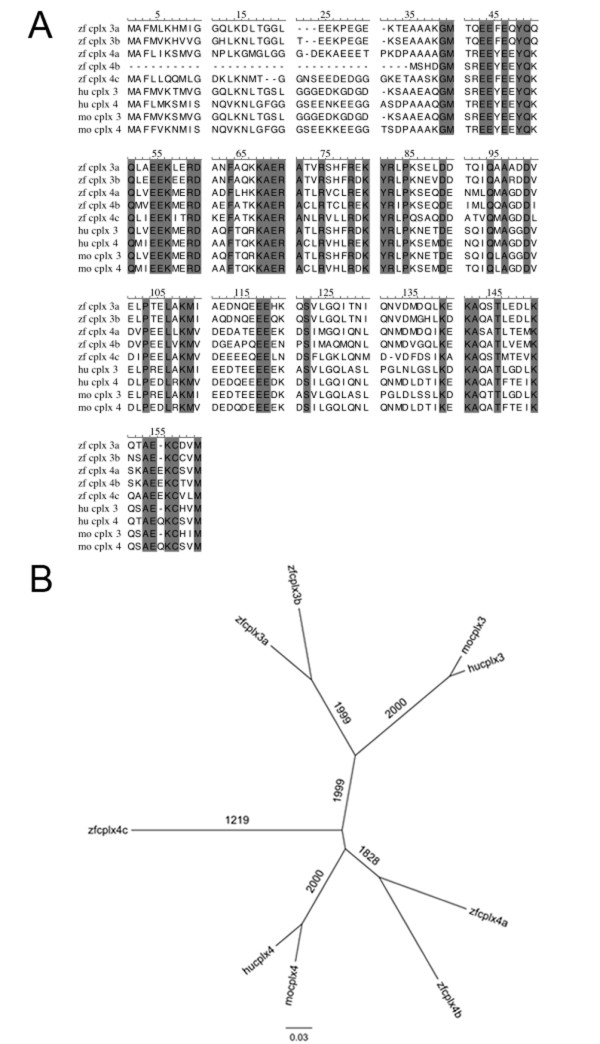
**The zebrafish genome contains two complexin 3 orthologs and three complexin 4 orthologs**. **(A) **The deduced amino acid sequences of mouse, human, and zebrafish complexins 3 and 4 are aligned with ClustalW. The shaded regions highlight evolutionarily conserved amino acids. Complexins 3 and 4 are highly homologous to each other (50 to 75% amino acid identity), but not to the complexin 1/2 subfamily (approximately 25% amino acid identity). Members of the complexin 3/4 subfamily share two motifs not present in the complexin 1/2 subfamily - a carboxy terminus prenylation motif (CAAX) and a putative bipartite nuclear localization signal (KKAERATVRSHFREKYRL in zebrafish complexin 3a). The GenBank accession numbers are: zf cplx 3a, [GenBank:GU174497]; zf cplx 3b, [GenBank:GU174498]; zf cplx 4a, [GenBank:GU174499]; zf cplx 4b, [GenBank:GU174500]; zf cplx 4c, [GenBank:GU174501]; hu cplx 3, [GenBank:NP_001025176]; hu cplx 4, [GenBank:NP_857637]; mo cplx 3, [GenBank:NP_666335]; mo cplx 4, [GenBank:NP_663468]. **(B) **An unrooted phylogram of vertebrate complexin 3 and 4 proteins was constructed with the neighbor joining method, and bootstrap values were calculated from 2,000 trials. Branch length is proportional to evolutionary divergence. Zebrafish complexins 3a and 3b segregate closely with mammalian complexin 3, and zebrafish complexins 4a and 4b segregate closely with mammalian complexin 4. A fifth paralog is designated zebrafish complexin 4c given its relatively closer distance to mammalian complexin 4 isoforms. Cplx, complexin; hu, human; mo, mouse; zf, zebrafish.

By searching the zebrafish ENSEMBL and GenBank sequence databases, we identified five zebrafish orthologs of mammalian complexins 3 and 4. The full-length coding sequences of each zebrafish ortholog were then amplified by PCR from retina cDNA. Translation of the putative open reading frames within the amplified PCR products predicted proteins that range, with one exception, from 155 to 159 amino acids in length (Figure 1A). The five zebrafish orthologs show approximately 50 to 75% amino acid identity with human and mouse complexins 3 and 4. Alignment of these proteins reveals striking amino acid identity adjacent to, and within, the putative core alpha helices (RDAQFTQRKAERATLRSHFRDKY in mouse complexin 3) that may mediate binding to SNARE complexes [23]. Members of the complexin 3/4 subfamily share two motifs not present in the complexin 1/2 subfamily: a prenylation motif (CAAX) at the carboxy terminus that is important for membrane targeting [23]; and a putative bipartite nuclear localization signal (RKAERATLRSHFRDKYRL in mouse complexin 3) that almost completely overlaps with the putative SNARE-binding core alpha helix. Taken together, the sequence identity and conserved structural features suggest a novel vertebrate subfamily consisting of complexin 3 and 4 isoforms.

To confirm that the zebrafish clones are orthologs of mammalian complexins 3 and 4, we analyzed their phylogenetic and syntenic relationships. The aligned amino acid sequences of zebrafish, human, and mouse complexin 3 and 4 orthologs were used to construct a phylogenetic tree (Figure 1B). While two zebrafish orthologs cluster with mammalian complexin 3, three zebrafish orthologs resemble mammalian complexin 4. The percent identity between the zebrafish complexin 3a protein and either the mouse or human complexin 3 is 63%, while the percent identity between zebrafish complexin 3b and either mouse or human complexin 3 is 67 to 68%. Zebrafish complexins 4a and 4b share more identity with mammalian complexin 4 (66 to 73%) than complexin 3 (53 to 57%). Zebrafish complexin 4c is the most divergent member of this subfamily, sharing 50% identity with either mouse or human complexin 3 and 57% identity with either mouse or human complexin 4. The five zebrafish orthologs of mammalian complexins 3 and 4 indicate the presence of several *complexin *(*cplx*) gene duplication events in the evolutionary history of this teleost.

**Figure 2:**
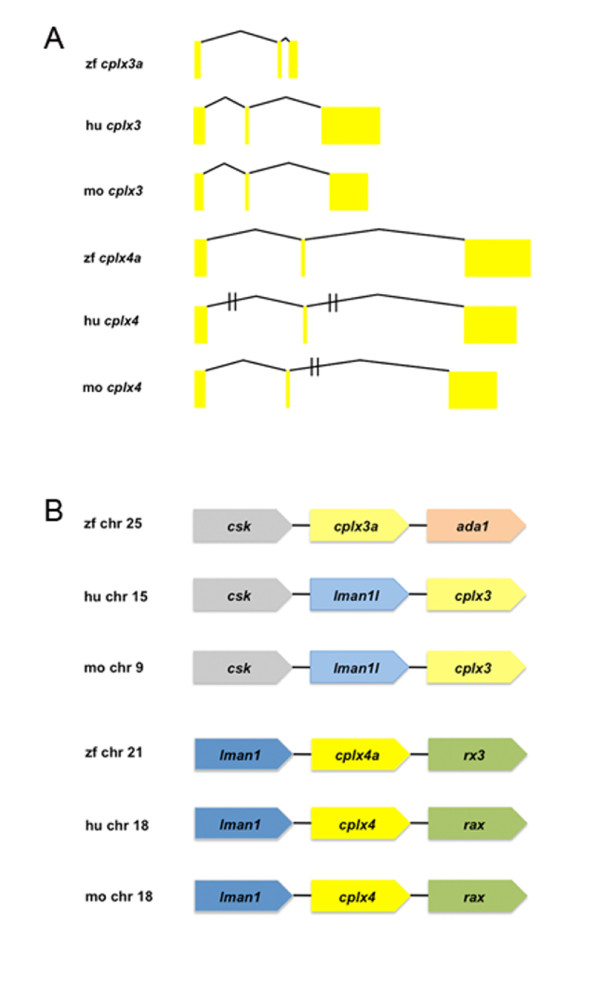
**Exon-intron organization of orthologous mammalian and zebrafish *cplx 3 *and *cplx 4 *genes and their syntenic relationships**. **(A) **The gene structures for human, mouse, and some zebrafish *cplx 3 *and *4 *orthologs are shown. Exons are denoted by yellow boxes, while introns are indicated with connecting lines. The lengths of the exons and introns are drawn to scale, except for human and mouse *cplx 4*. *Cplx 3*/*4 *gene structure is highly conserved, with three exons interrupted by two introns. The length of the second exon, which encodes part of the putative SNARE-binding domain, is exactly 88 nucleotides in all species examined. **(B) **Syntenic relationships of zebrafish, human, and mouse *cplx 3*/*4 *orthologs are shown. The genomic architectures of mammalian *cplx 3 *orthologs and mammalian *cplx 4 *orthologs are conserved. Human and mouse *cplx 3 *are preceded by *CSK *and *LMAN1L*/*ERGIC-53L*, while human and mouse *cplx 4 *are bounded by *LMAN1*/*ERGIC-53 *and *RAX*. Zebrafish *cplx 3a*, which has been mapped to chromosome 25, is preceded by a zebrafish ortholog of *CSK*. Zebrafish *cplx 4a *is bordered by *LMAN *and *RX3*, which is the zebrafish ortholog of mammalian *RAX*. Ada, adenosine deaminase; chr, chromosome; cplx, complexin; CSK, c-src kinase; ERGIC-53, ER Golgi intermediate compartment 53-kDa; ERGIC-53L, ERGIC-53, ER Golgi intermediate compartment 53-kDa-like; hu, human; LMAN1, lectin mannose-binding 1; LMAN1l, lectin mannose-binding 1-like; mo, mouse; RAX, retina and anterior neural fold homeobox gene; rx3, retinal homeobox gene 3; zf, zebrafish.

The gene structure for vertebrate *cplx 3*/*4 *subfamily members appears highly conserved, with three exons separated by two introns (Figure 2A). The second exon, which contains the sequence that encodes the putative core helix of the SNARE-binding domain, has an invariant length of 88 nucleotides in all examined *cplx 3*/*4 *orthologs. This strict preservation of exon size throughout evolution underscores an important functional role for this module. Furthermore, these results suggest that *cplx 3 *and *cplx 4 *resulted from the duplication of a common ancestor.

In potential support of this hypothesis, synteny analysis reveals conserved relationships between genes surrounding mammalian *cplx 3 *and *cplx 4 *and some of their zebrafish orthologs. For example, the *lectin mannose-binding 1 *(*LMAN1*)/*ER Golgi intermediate compartment 53-kD *(*ERGIC-53*) and *retina and anterior neural fold homeobox *(*RAX*) genes surround *cplx 4 *on human and mouse chromosome 18 (Figure 2B). Similarly, *cplx 4a *on zebrafish chromosome 21 is surrounded by orthologs of *LMAN1*/*ERGIC-53 and RAX. *A gene in the *LMAN1*/*ERGIC-53 *family, called *LMAN1L*/*ERGIC-53L*, and the *CSK tyrosine kinase *are situated 5' to *cplx 3 *on human chromosome 15 and mouse chromosome 9. A c*-src kinase *(*CSK*) ortholog is present 5' to *cplx 3a *on zebrafish chromosome 25, but an *LMAN1L*/*ERGIC-53L *ortholog has not yet been detected in the zebrafish genome. The presence of *LMAN1*/*ERGIC-53 *or *LMAN1L*/*ERGIC-53L *immediately 5' to all examined mammalian *cplx 3*/*4 *genes, as well as zebrafish *cplx 4a*, suggests an ancestral unit composed of an *LMAN*/*LMAN1-L *family member and a *cplx 3*/*4*. Based on these phylogenetic and syntenic relationships, as well as conservation of gene and protein architecture, we conclude that zebrafish contain five orthologs of the complexin 3/4 subfamily.

### Enrichment of complexin 3/4 in visual system ribbon terminals in larval zebrafish

Previous studies have revealed that complexins 3 and 4 are expressed in the adult mammalian retina, primarily in ribbon presynaptic terminals [23,28]. A polyclonal antibody raised against mouse complexin 3 labeled cone photoreceptor and rod bipolar cell terminals, while a polyclonal antibody directed against mouse complexin 4 labeled rod photoreceptor and cone bipolar cell terminals [23]. To determine whether these antibodies recognize the zebrafish complexin 3/4 orthologs, we transfected HEK 293T cells (which do not endogenously express complexins 3 or 4; data not shown) with individual myc-tagged, full-length complexins and performed immunocytochemistry with the polyclonal antibodies. In parallel, we visualized expression of the fusion proteins with a monoclonal anti-myc antibody. Additional file 1 contains micrographs of representative cells transfected with complexin 3a (Additional file 1 A,B), complexin 3b (Additional file 1C,D), complexin 4a (Additional file 1E,F), complexin 4b (Additional file 1G,H), and complexin 4c (Additional file 1I,J) and stained with the polyclonal antibody raised against mouse complexin 3 (Synaptic Systems antibody 122302) (Additional file 1A,C,E,G,I) and the anti-myc monoclonal (Additional file 1B,D,F,H,J). These results demonstrate that this polyclonal antibody recognizes all five zebrafish orthologs at a dilution of 1:10,000 (the dilution used in [23] to stain mouse retina), and are quantified in Additional file 1K. HEK 293T cells transfected with the expression vector did not exhibit any immunoreactivity when incubated with the complexin polyclonal antibody (data not shown). This antibody detects predominantly a 20 kDa band on western blots of adult mouse and zebrafish retina, together with a smaller band as expected for complexin 4b, indicating that the mouse complexin antibody recognizes proteins of the predicted molecular weight (Additional file 2).

**Figure 3:**
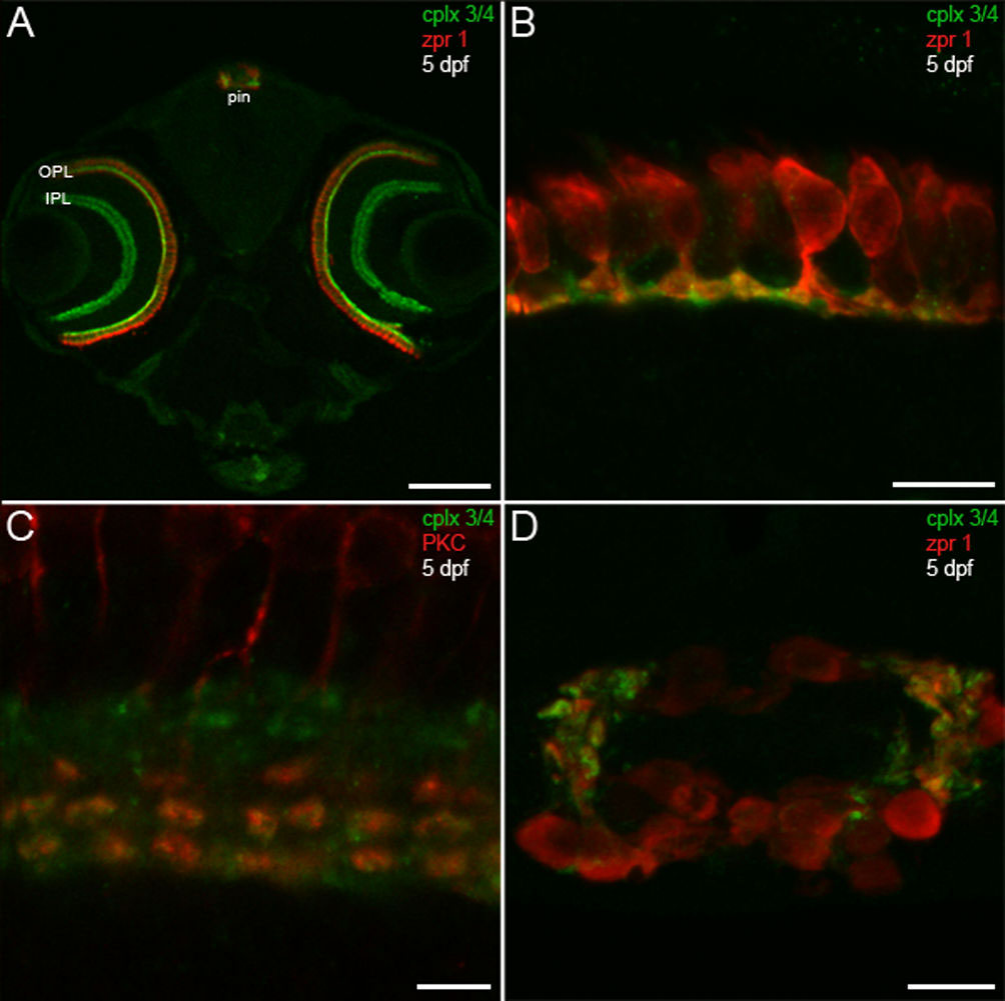
**Predilection of complexin 3/4 for ribbon presynaptic terminals in the larval zebrafish visual system**. **(A) **A confocal projection of a 5-dpf zebrafish transverse section stained with the pan-complexin 3/4 antibody (green) and the zpr 1/FRet 43 antibody (red), which labels double cone photoreceptors, reveals complexin 3/4 immunoreactivity throughout the retinal plexiform layers (OPL, IPL) and in the pineal organ (pin). Complexin 3/4 is absent from the retinal nuclear layers and the medial region of the pineal organ, which contains photoreceptor somata. **(B) **A high-magnification confocal projection of double cone photoreceptors (red) in the retinal OPL labeled with the complexin 3/4 antibody (green) shows that some of the complexin 3/4 immunoreactivity in the larval zebrafish OPL is found in double cone terminals. **(C) **Double-labeling with anti-protein kinase C (red) reveals overlap of complexin 3/4 (green) in ON bipolar cell terminals in the retinal IPL. **(D) **A confocal projection of a transverse section through the pineal organ reveals zpr 1/FRet 43-positive photoreceptors (red) and complexin 3/4 (green) in processes and terminals at the lateral border. Sections incubated with secondary antibodies alone exhibit background immunofluorescence in the retina and pineal (data not shown). Scale bars: 125 μm (A); 10 μm (B, D); 5 μm (C).

The pan-complexin 3/4 antibody was then used to examine the expression of this subfamily in the larval zebrafish nervous system. Sensory systems develop rapidly in zebrafish such that visual and acousticolateral responses can be robustly elicited by five days post-fertilization (dpf) [29,30]. Therefore, 5-dpf larval sections were stained with the pan-complexin 3/4 antibody and the zpr 1/FRet 43 monoclonal antibody, which is a marker for double cone photoreceptors in zebrafish [31]. As in adult mammals [23], complexin 3/4 is most highly expressed in the retina in larval zebrafish (Figure 3A). Striking complexin 3/4 immunoreactivity can be observed throughout the retinal OPL and IPL, but not in the outer nuclear layer, inner nuclear layer, or ganglion cell layer (Figure 3A). Complexin 3/4 co-localizes with zpr 1/FRet 43 in terminals of double cone photoreceptors, but is not restricted to these terminals in the OPL (Figure 3B). In the IPL, complexin 3/4 appears in both sublamina a and sublamina b, overlapping in the latter with protein kinase C in ON bipolar cell terminals [32] (Figure 3C).

Besides the retina, the pineal complex is photosensitive in sauropsids and anamniotes (reviewed in [2]). In the zebrafish pineal organ, ribbon-containing photoreceptors send short axons ventrolaterally to terminate on projection neurons [33,34]. Complexin 3/4 concentrates in zpr 1/FRet 43-positive processes and terminals at the lateral border of the pineal organ (Figure 3D). Additional complexin 3/4 immunoreactivity can be observed in zpr 1/FRet 43-negative processes in the pineal neuropil (Figure 3D), which is a plexus that contains processes from several types of photoreceptors (reviewed in [2]). Taken together, these results indicate that complexin 3/4 delineates many ribbon-containing sensory neurons in the larval zebrafish photoreceptive organs.

### Concomitant targeting of complexin 3/4 and RIBEYE b to photoreceptor terminals during zebrafish embryogenesis

**Figure 4:**
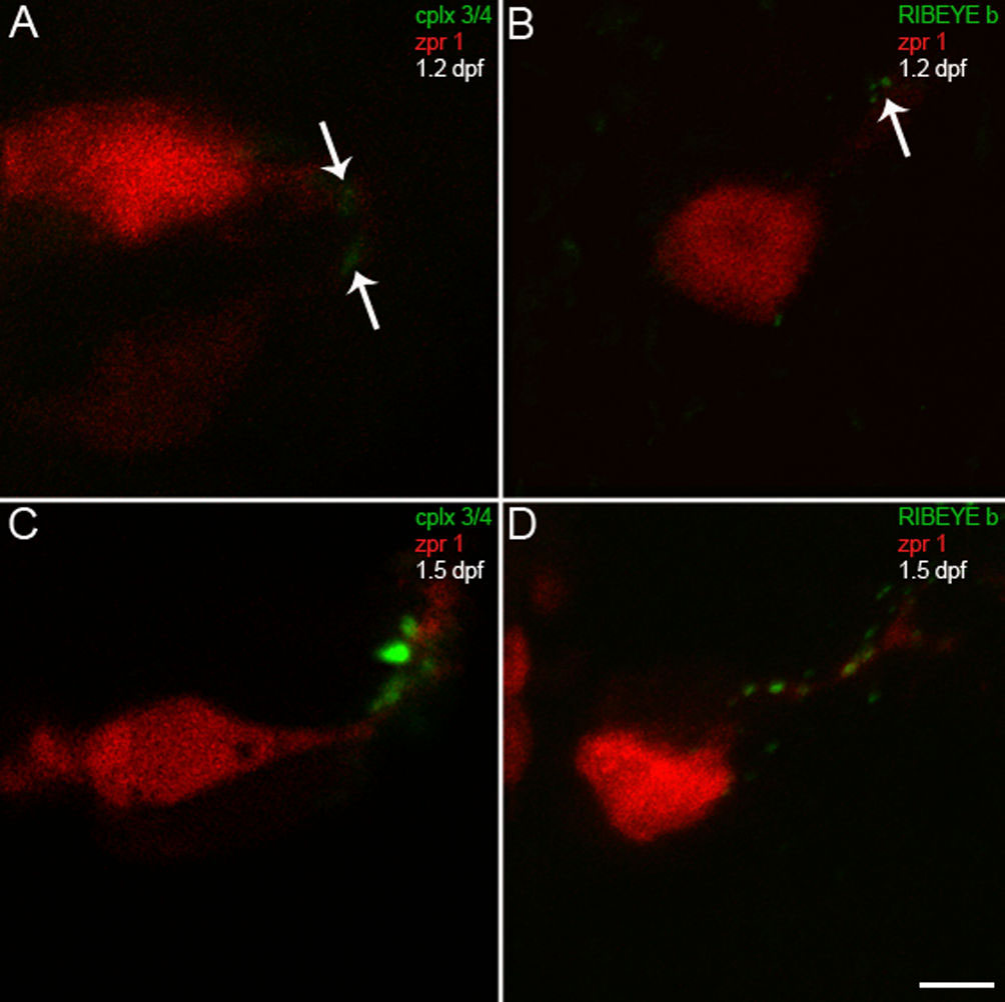
**Complexin 3/4 concentrates in pineal photoreceptor terminals concomitant with RIBEYE b**. **(A) **A high-magnification confocal projection of zpr 1/FRet 43-positive pineal double cone photoreceptors (red) double-stained for complexin 3/4 (green) at 1.2 dpf shows low levels of complexin in photoreceptor terminals (arrows). Ventral is toward the top and lateral is toward the right. **(B) **A section from a different embryo at 1.2 dpf double-stained with anti-zpr 1/FRet 43 (red) and anti-RIBEYE b (green) shows three small RIBEYE puncta in a pineal photoreceptor terminal (arrow). **(C) **By 1.5 dpf, complexin 3/4 (green) is highly expressed in neuropil at the lateral border of the pineal organ, in both zpr 1/FRet 43-positive (red) and zpr 1/FRet 43-negative photoreceptor axons and terminals. **(D) **Several RIBEYE b puncta (green) are present in pineal photoreceptor axons and terminals at 1.5 dpf. Scale bar = 5 μm.

To begin to characterize the targeting of the exocytotic machinery to ribbon terminals, we examined the spatiotemporal expression profiles of complexin 3/4 and RIBEYE b relative to zpr 1/FRet 43 during photoreceptor development. In teleosts, photoreceptors differentiate in the pineal organ before differentiating in the retina. In zebrafish, most pineal progenitors exit the cell cycle between 18 and 20.5 hours post-fertilization (hpf) [35]. Therefore, embryonic zebrafish were fixed at several developmental time points starting at 1 dpf. No complexin 3/4 or zpr 1/FRet 43 immunoreactivity was apparent in the pineal organ at 1 dpf (data not shown). At 1.2 dpf, small numbers of zpr 1/FRet 43-positive photoreceptors with short axons can be observed (Figure 4A, red). Very low levels of complexin 3/4 are found in zpr 1/FRet 43-positive pineal photoreceptors, and the immunoreactivity localizes to the developing neuropil (Figure 4A, green, arrows). To examine the targeting of other components of the exocytotic machinery, we utilized a polyclonal antibody directed against RIBEYE b [36]. At 1.2 dpf, RIBEYE b is also found at very low levels in zpr 1/FRet 43-positive pineal photoreceptor terminals (Figure 4B, arrow). By 1.5 dpf, the neuropil at the lateral border of the pineal has become well-developed, with processes emanating from dorsomedial zpr 1/FRet 43-positive and zpr 1/FRet 43-negative photoreceptor somata. Complexin 3/4 expression has been upregulated in processes and terminals, some of which co-localizes with zpr 1/FRet 43 (Figure 4C). Prominent RIBEYE b puncta are found in photoreceptor axons and their terminals (Figure 4D).

**Figure 5:**
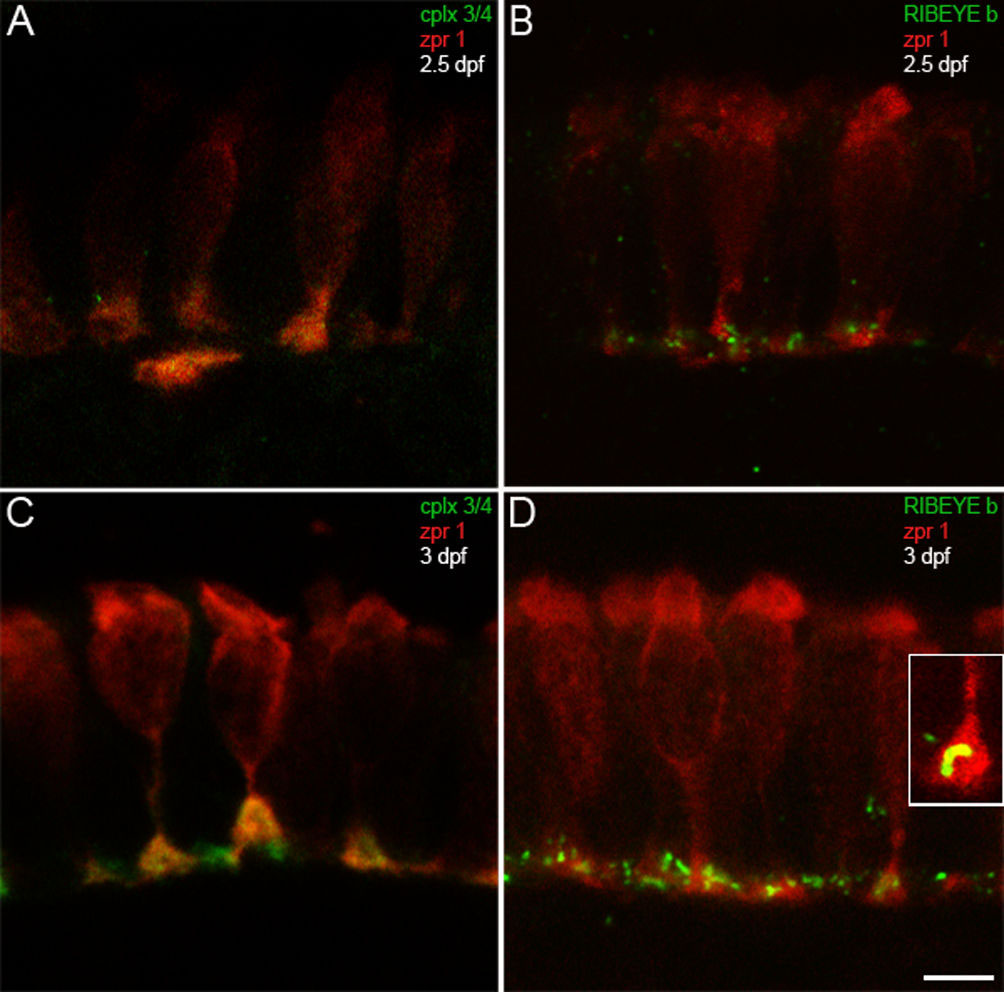
**Complexin 3/4 concentrates in retinal photoreceptor terminals concomitant with RIBEYE b**. **(A) **The zpr 1/FRet 43 monoclonal antibody (red) and the pan-complexin 3/4 polyclonal (green) were also used to localize these complexins in developing retinal photoreceptors. At 2.5 dpf, retinal photoreceptors are most prominent in the ventronasal patch, where complexin 3/4 immunoreactivity appears in some photoreceptor terminals. **(B) **RIBEYE b (green) has also started to cluster in photoreceptor terminals (red) in the outer plexiform layer at 2.5 dpf. **(C) **At 3 dpf, complexin 3/4 (red) is highly expressed in zpr 1/FRet 43-positive (red) and zpr 1/FRet 43-negative photoreceptor terminals in the ventronasal patch. **(D) **Pleiomorphic RIBEYE b (green) expression is found at 3 dpf in zpr 1/FRet 43-positive (red) and zpr 1/FRet 43-negative photoreceptor terminals in the ventronasal patch. The inset shows a zpr 1/FRet 43-positive terminal containing curvilinear RIBEYE b immunoreactivity that may correspond to a ribbon. Scale bar = 5 μm.

We next examined complexin 3/4 expression in the developing retina. At 2 dpf, zebrafish photoreceptors are exiting the cell cycle [37], and small numbers of zpr 1/FRet 43-positive photoreceptors can be observed in the retinal ventronasal patch at 50 hpf [38]. At 2 dpf, we did not observe zpr 1/FRet 43 in the retina (data not shown). By 2.5 dpf, zpr 1/FRet 43-positive photoreceptors are found in the ventronasal patch, where they express complexin 3/4 (Figure 5A) and RIBEYE b (Figure 5B) in their terminals. By 3 dpf, complexin 3/4 (Figure 5C, green) and RIBEYE b (Figure 5D, green) have become concentrated in photoreceptor terminals throughout the retina. In some terminals, RIBEYE b has clustered into curvilinear structures (Figure 5D, inset) that most likely correspond to ribbons [39]. Taken together, these results suggest that complexin 3/4 and RIBEYE b appear concomitantly in retinal and pineal photoreceptors and are rapidly targeted to their axons and terminals.

### Complexin 3/4 immunoreactivity on the apical surfaces of inner ear and lateral line hair cells in larval zebrafish

**Figure 6:**
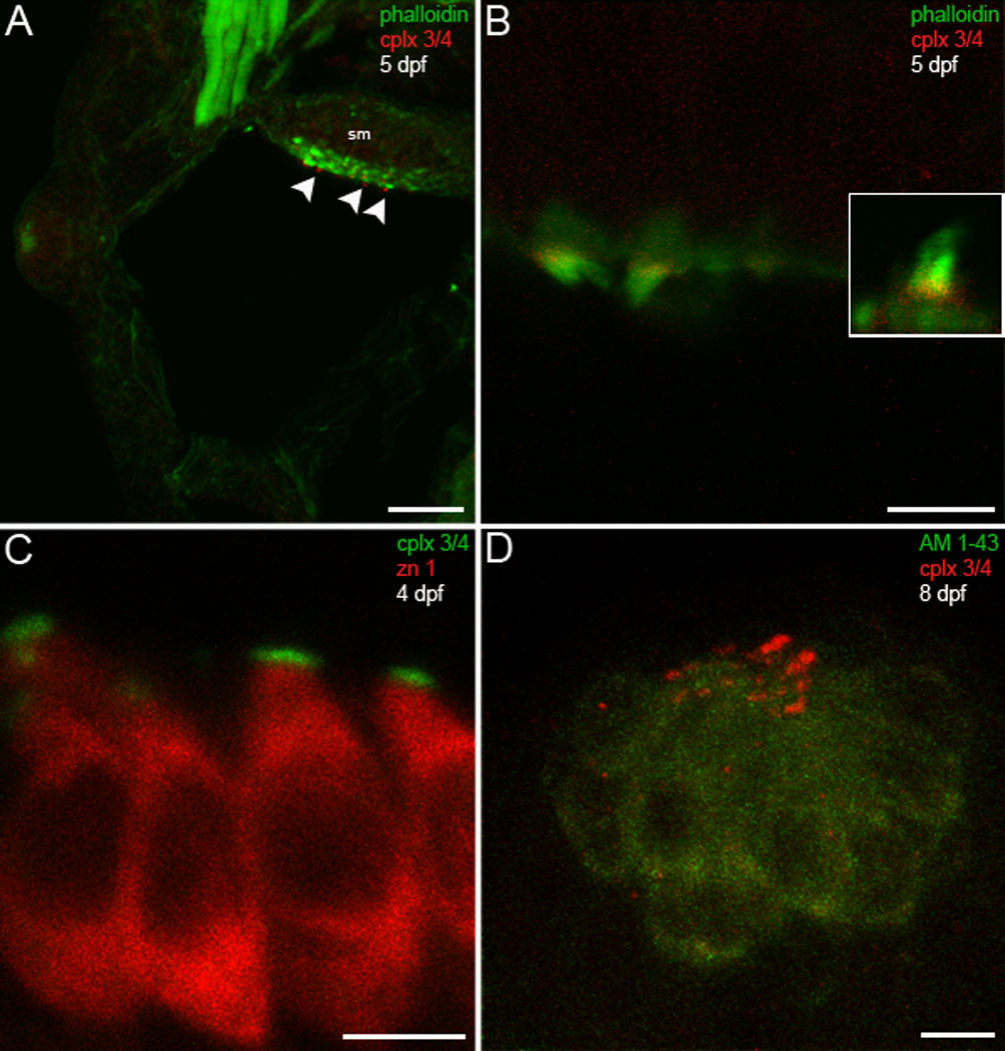
**Hair cells in the larval zebrafish inner ear and lateral line exhibit complexin 3/4 immunoreactivity on their apical surfaces**. **(A) **A confocal projection of a 5-dpf zebrafish transverse section through the otic vesicle reveals complexin 3/4 immunoreactivity (red) on the apical surfaces of inner ear hair cells in the saccular macula (sm; arrowheads) labeled with phalloidin (green). **(B) **A high-magnification en face view shows complexin 3/4 immunoreactivity (red) at the base of stereocilia labeled by phalloidin above the actin-rich cuticular plate. The inset contains an enlargement of a hair bundle. **(C) **Complexin 3/4 immunoreactivity (green) directly abuts zn 1 cytoplasmic immunoreactivity (red) on the apical surfaces of inner ear hair cells. **(D) **To examine neuromast hair cells more closely, larval zebrafish were incubated with AM1-43 (green), fixed, sectioned sagittally, and labeled with anti-complexin 3/4 (red). Complexin 3/4 immunoreactivity is also present on the apical surfaces of neuromast hair cells. Neuromast and inner ear sections incubated with secondary antibodies alone exhibit background immunofluorescence (data not shown). Scale bars: 25 μm (A); 5 μm (B-D).

To determine whether members of the complexin 3/4 subfamily are also expressed in sensory hair cells, we compared the distribution of complexin 3/4 immunoreactivity with that of fluorophore-tagged phalloidin, which labels actin-rich stereociliary bundles. In the larval zebrafish inner ear, striking complexin 3/4 immunoreactivity appears among the stereociliary bundles, but not in the basolateral domains, of macular hair cells (Figure 6A, arrowheads). Complexin 3/4 also localizes to stereociliary bundles in hair cells of the anterior, lateral and posterior cristae (data not shown). At higher magnification, it becomes evident that complexin 3/4 immunoreactivity concentrates at the base of the stereociliary bundle (Figure 6B). Double-labeling with anti-zn 1, a monoclonal antibody that recognizes a cytoplasmic antigen found in subpopulations of hair cells [40,41], confirms that complexin 3/4 localizes to the apical surface of the hair cell (Figure 6C). Substantial accumulation of complexin 3/4 immunoreactivity was also observed on the apical surfaces of hair cells in the neuromasts of the cranial (Figure 6D) and trunk lateral lines (data not shown). In summary, these results indicate that members of the complexin 3/4 subfamily are enriched in many sensory neurons that contain ribbons. Furthermore, complexin 3/4 isoforms localize to distinct subcellular domains in different sensory systems.

### Rapid clustering of complexin 3/4 immunoreactivity on the apical surfaces of inner ear and lateral line hair cells in embryonic zebrafish

**Figure 7:**
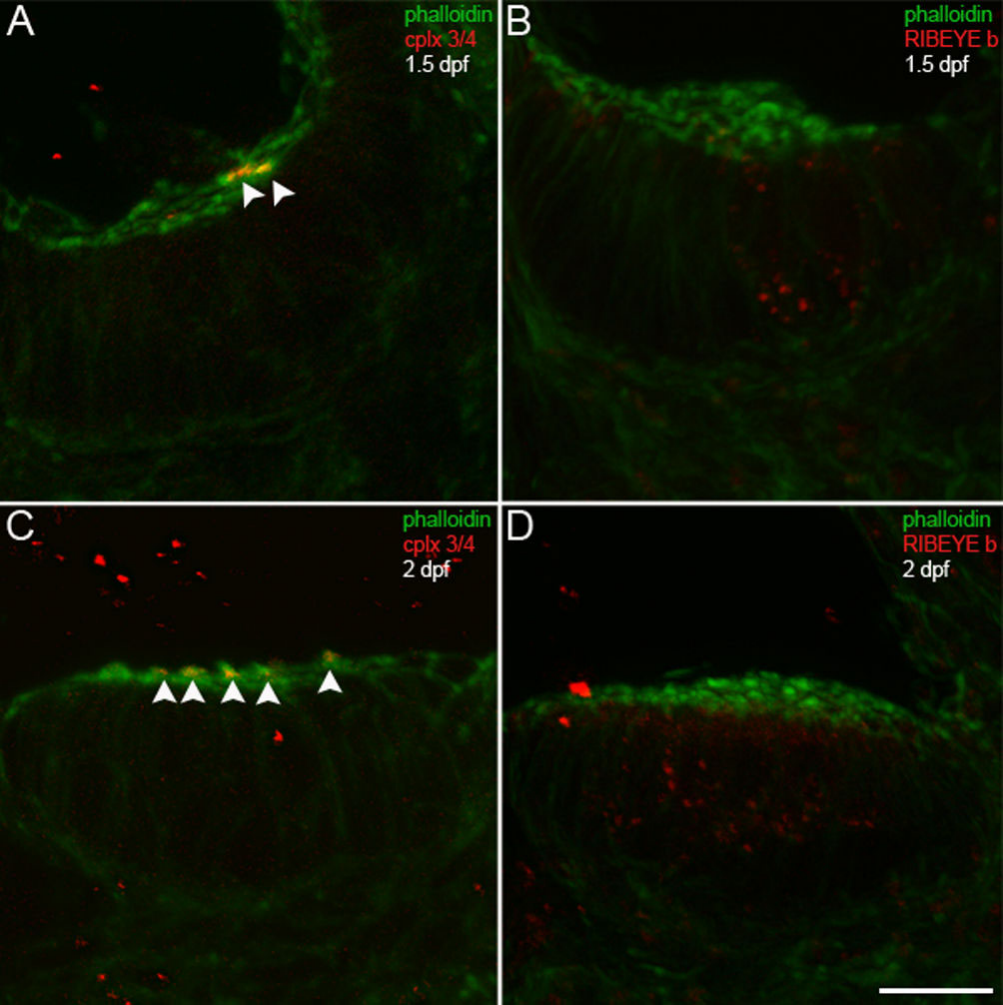
**Complexin 3/4 does not co-localize with RIBEYE b in embryonic inner ear hair cells**. **(A) **A high-magnification confocal projection through the posterior macula in the zebrafish inner ear at 1.5 dpf. Using phalloidin (green) as a marker of developing hair cell stereocilia, one can observe a small number of hair cells with stereocilia. Complexin 3/4 (red) is already apparent among these stereocilia (arrowheads). **(B) **Dual immunolabeling of RIBEYE b (red) and F-actin with phalloidin (green) in the anterior macula at 1.5 dpf. Note that RIBEYE b has clustered into large puncta on the basolateral membrane of hair cells with stereocilia. **(C) **At 2 dpf, many more hair cells with stereocilia (green) can be observed in the anterior macula. Complexin 3/4 immunoreactivity (red) can be observed among these stereocilia (arrowheads). **(D) **RIBEYE b (red) is upregulated in the cytoplasm of more hair cells in the anterior macula at 2 dpf. Scale bar = 10 μm.

Hair cells in the zebrafish inner ear [42,43] and lateral lines [44] differentiate rapidly, with formation of stereocilia soon after exit from the cell cycle. The first hair cells to differentiate, called tether cells, exist in two pairs that project stereocilia and kinocilia into the otic vesicle by 1 dpf [42,43]. We have observed putative tether cells with complexin 3/4 immunoreactivity solely among their stereocilia at 1 dpf (data not shown). At 1.5 dpf, few hair cells in the anterior and posterior maculae have stereocilia, but those that do typically have complexin 3/4 immunoreactivity primarily on their apical surfaces (Figure 7A, red, arrowheads) and RIBEYE b puncta on their basolateral surfaces (Figure 7B, red). The latter finding is consistent with a recent report showing RIBEYE b at the base of inner ear hair cells at 27 hpf [45]. By 2 dpf, several macular hair cells have acquired actin-rich stereocilia with complexin 3/4 (Figure 7C, arrowheads) and have upregulated RIBEYE b (Figure 7D, red).

**Figure 8:**
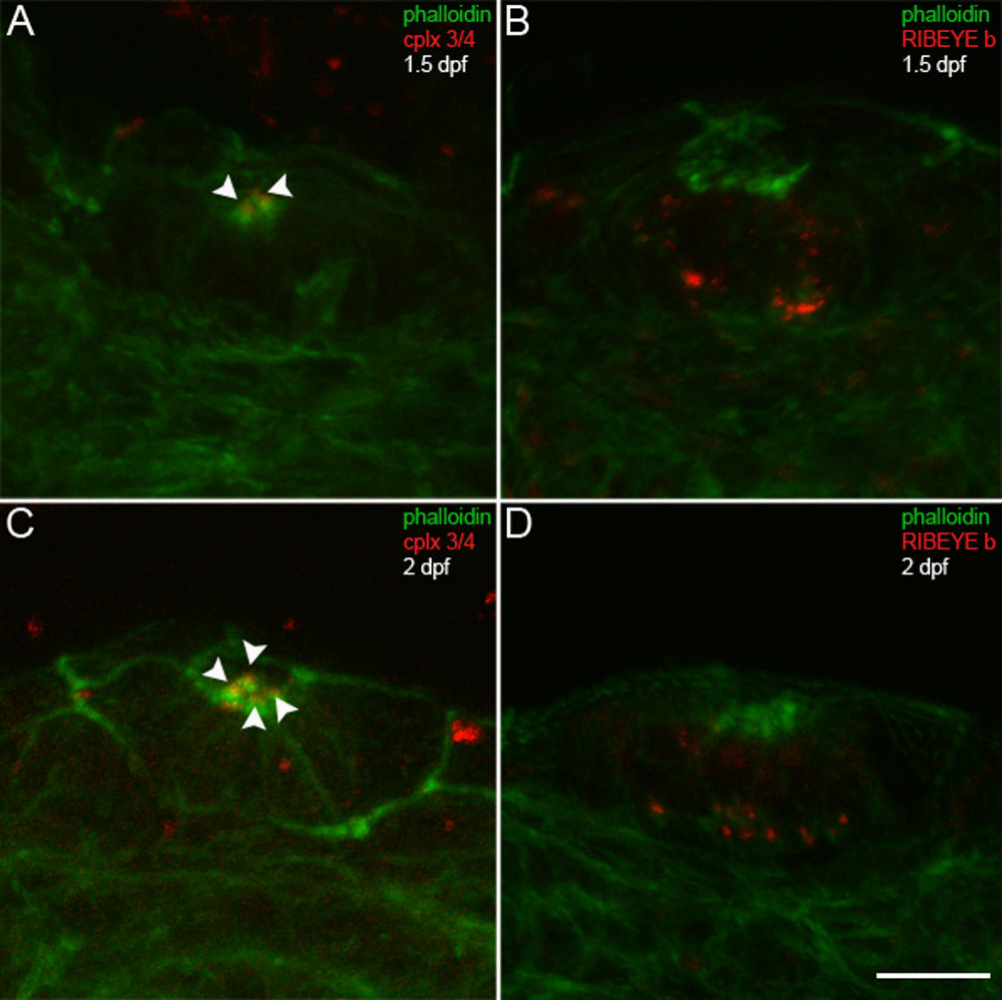
**Complexin 3/4 does not co-localize with RIBEYE b in embryonic neuromast hair cells**. **(A) **Embryonic zebrafish at 1.5 dpf were sectioned transversely. Phalloidin (green) and anti-complexin 3/4 (red) were used to probe otic neuromasts. Note that complexin 3/4 is weakly expressed among neuromast hair cell stereocilia at this time (arrowheads). **(B) **Large RIBEYE b puncta (red) can be observed at the base, and diffuse cytoplasmic immunoreactivity elsewhere, in a couple of neuromast hair cells at 1.5 dpf. **(C) **Greater numbers of hair cells in an otic neuromast have stereocilia (green) at 2 dpf. These hair cells also have apical complexin 3/4 immunoreactivity (red, arrowheads). **(D) **At 2 dpf, many hair cells in an otic neuromast have RIBEYE b immunoreactivity (red). Scale bar = 10 μm.

In the zebrafish lateral lines, the otic neuromast differentiates first at 34 hpf [46]. At 1.5 dpf, phalloidin labels otic neuromast hair cell stereocilia with faint complexin 3/4 immunoreactivity (Figure 8A, arrowheads). By this time, RIBEYE b has already concentrated in large puncta at the base, and in smaller cytoplasmic puncta, in a couple of hair cells in the neuromast (Figure 8B, red). By 2 dpf, complexin 3/4 (Figure 8C, red, arrowheads) and RIBEYE b (Figure 8D, red) have been upregulated in more hair cells in the otic neuromast. Ultrastructural evidence of mature ribbons in zebrafish neuromast hair cells at 2 dpf has previously been reported [44]. Taken together, these results suggest that complexin 3/4 and RIBEYE b immunoreactivities are targeted rapidly to different compartments in zebrafish inner ear and lateral line hair cells.

### Complexin 4a specifically marks visual system ribbon presynaptic terminals

To determine the immunoreactivity profile for the polyclonal antibody directed against mouse complexin 4 [23], we performed the immunofluorescence assay with the transfected HEK 293T cells that express individual complexin 3/4 isoforms. As can be seen in Additional file 3, the complexin 4 polyclonal antibody (Synaptic Systems antibody 122402; when used at a dilution of 1:75,000) preferentially recognizes zebrafish complexin 4a. Additional file 3B,D,F,H,J show representative HEK 293T cells transfected with myc-tagged, full-length complexins 3a, 3b, 4a, 4b, and 4c, respectively, and labeled with the anti-myc monoclonal antibody. Additional file 3A,C,E,G,I contain the same cells incubated with the complexin 4 polyclonal antibody. The myc-tagged complexin 4a cell line (Additional file 3E,F) exhibits strong immunoreactivity with the complexin 4 antibody (Additional file 3E). These results are quantified in Additional file 3K and demonstrate that the polyclonal antibody directed against mouse complexin 4 is relatively selective for complexin 4a in zebrafish.

We have confirmed the findings of [23] that, in rodent retina, the polyclonal antibody directed against mouse complexin 4 labels small terminals, suggestive of rod spherules, in a wide band in the OPL and small terminals in a thin band of the IPL (Additional file 4B). Furthermore, the polyclonal antibody directed against mouse complexin 3 labels large cone pedicles in the OPL and many terminals of the IPL (Additional file 4A, and see [23]).

**Figure 9:**
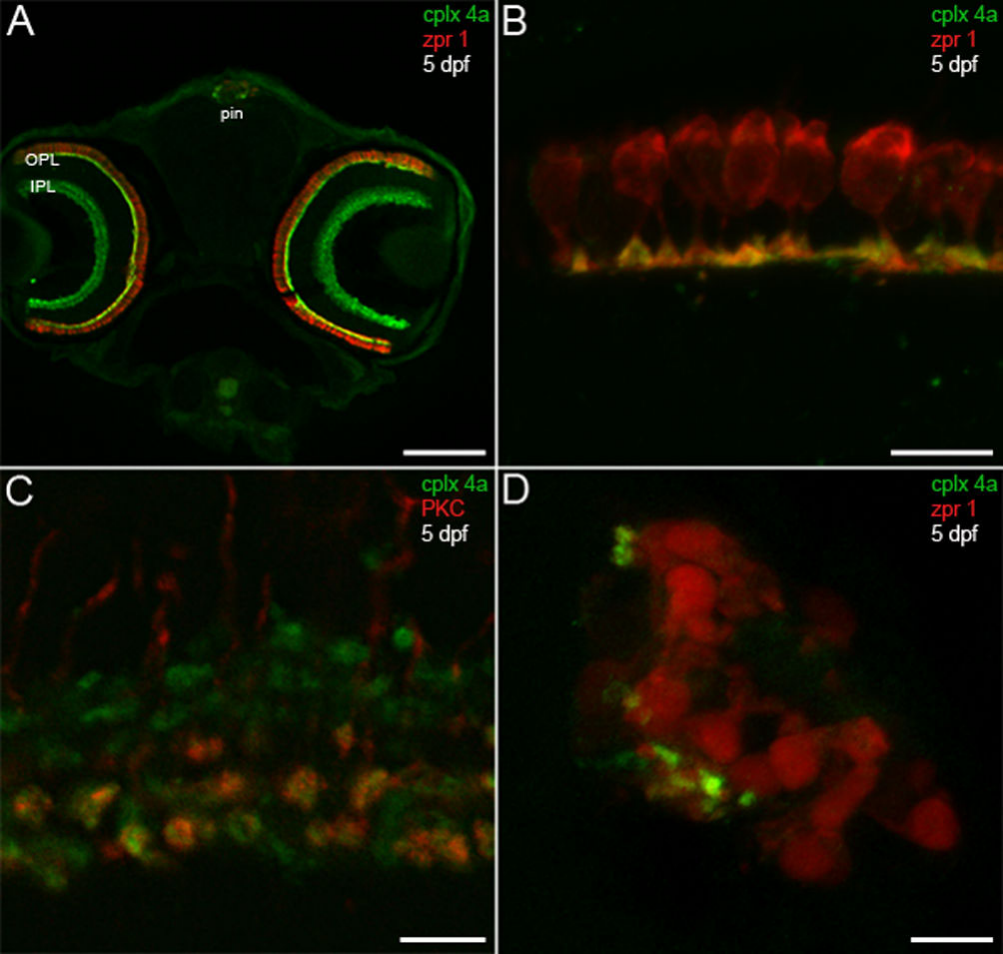
**Complexin 4a marks visual system ribbon presynaptic terminals**. **(A) **A confocal micrograph of a 5-dpf zebrafish transverse section stained with the anti-complexin 4a antibody (green) and anti-zpr 1/FRet 43 (red). Note the similarity between the complexin 4a expression pattern and that of complexin 3/4 (Figure 3A) at low magnification in the retinal plexiform layers (OPL, IPL) and pineal organ (pin). **(B) **At high magnification, complexin 4a appears to be restricted primarily to the terminals of double cone photoreceptors in the retinal outer plexiform layer. **(C) **A confocal planar projection of the retinal inner plexiform layer shows overlap of complexin 4a (green) in protein kinase C (PKC)-positive (red) ON bipolar cell terminals in the IPL. **(D) **A confocal planar projection of a 5-dpf zebrafish sagittal section through the pineal organ is shown. Zpr 1/FRet 43-positive photoreceptors (red) are oriented such that their outer segments are medial and their short axons and presynaptic terminals are lateral. Complexin 4a (green) specifically localizes to putative terminals. Sections incubated with secondary antibodies alone exhibit background immunofluorescence in the retina and pineal (data not shown). Scale bars: 125 μm (A); 10 μm (B, D); 5 μm (C).

Staining of larval zebrafish sections with the complexin 4a antibody and anti-zpr 1/FRet 43 reveals abundant expression of complexin 4a in the retinal plexiform layers and lateral borders of the pineal organ (Figure 9A). Complexin 4a appears to be more restricted to the presynaptic terminals of retinal double cone (Figure 9B) and pineal photoreceptors (Figure 9D) when compared to pan-complexin 3/4 immunoreactivity in these cell types (Figure 3B,D). In the retinal IPL, complexin 4a is detectable in sublamina a and in protein kinase C-positive presynaptic terminals in sublamina b (Figure 9C).

**Figure 10:**
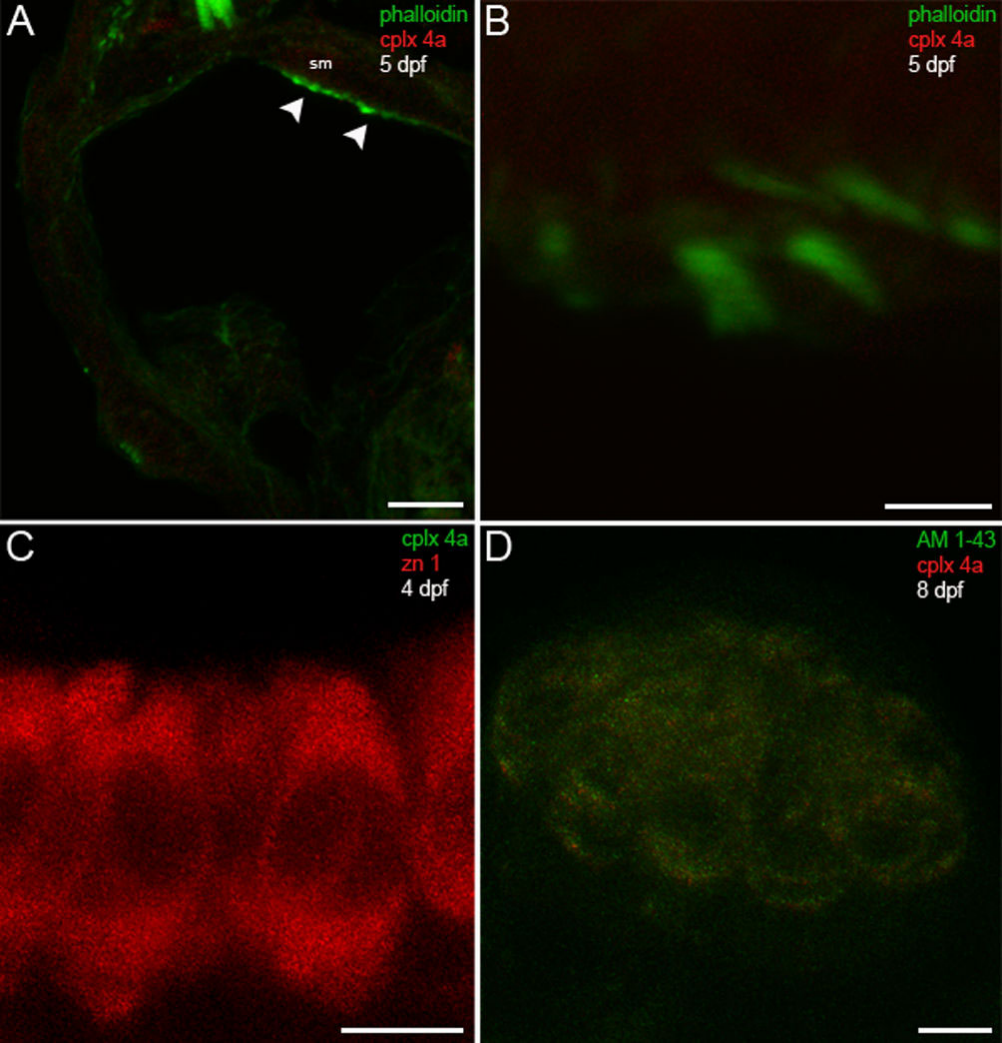
**Complexin 4a is not expressed in hair cells of the larval zebrafish acousticolateral system**. **(A) **A low-magnification confocal micrograph of a transverse section through the otic vesicle of a 5-dpf zebrafish shows saccular macula (sm) inner ear hair cell stereocilia stained with phalloidin (green) that lack complexin 4a (red, arrowheads). **(B) **A high-magnification en face view of inner ear hair cells labeled with phalloidin (green) confirms the absence of complexin 4a (red) among their stereocilia. **(C) **Inner ear hair cells incubated with anti-zn 1 (red) lack complexin 4a (green). **(D) **A high-magnification confocal planar projection through a cranial neuromast labeled with AM1-43 (green) indicates that complexin 4a is also absent from these hair cells. Sections incubated with only secondary antibodies exhibit very low levels of diffuse immunofluorescence throughout the hair cells (data not shown). Scale bars: 25 μm (A); 5 μm (B-D).

In contrast to the pan complexin 3/4 antibody, the complexin 4a antibody does not label hair cells in the inner ear or lateral line (Figure 10). Figure 10A shows a confocal planar projection of a larval zebrafish inner ear probed with phalloidin (green), to identify hair cells, and with the complexin 4a polyclonal antibody (red). Hair cells of the inner ear do not contain complexin 4a immunoreactivity (Figure 10A, arrowheads). Higher magnification of individual hair cells and their stereocilia confirms the absence of complexin 4a (Figure 10B). Inner ear sensory patches defined by the zn1 monoclonal antibody also lack complexin 4a (Figure 10C). Hair cells in neuromasts of the cranial (Figure 10D) and trunk (data not shown) lateral lines also lack complexin 4a. Taken together, these results indicate that complexin 4a is differentially expressed in ribbon-containing sensory neurons with a predilection for presynaptic terminals in the zebrafish visual system. In addition, hair cells contain one or more isoforms of the complexin 3/4 subfamily distinct from complexin 4a.

**Figure 11:**
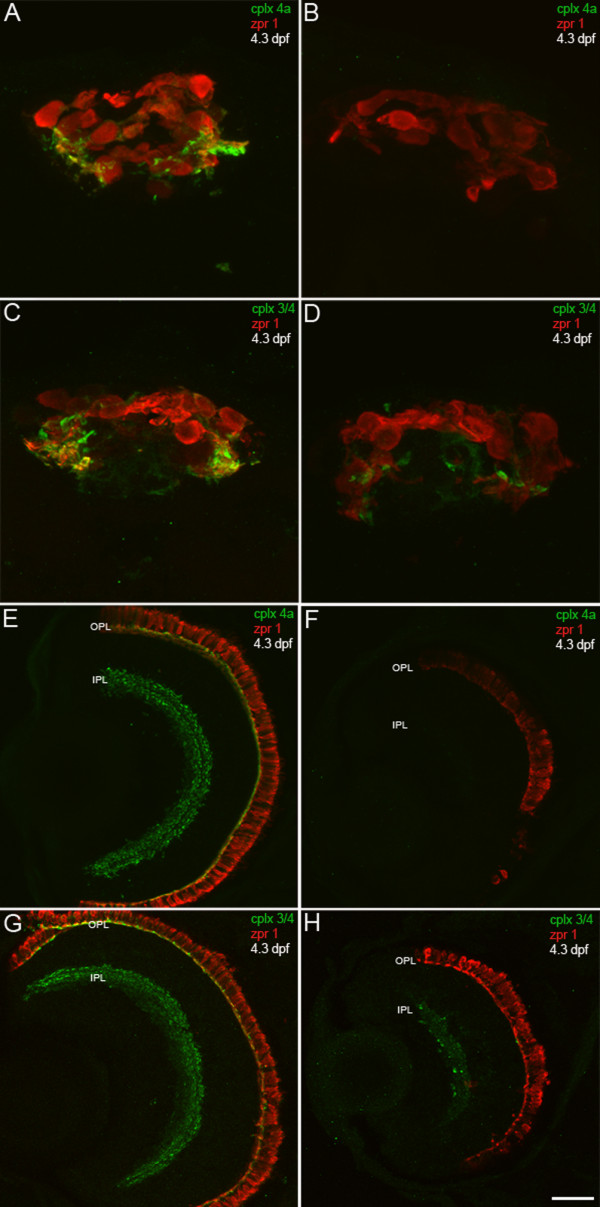
**A complexin 4a morpholino antisense oligonucleotide knocks down complexin 4a protein expression in the larval zebrafish retina and pineal organ**. **(A-H) **4.3-dpf control (A, C, E, G) and complexin 4a morpholino-injected (B, D, F, H) larval zebrafish were sectioned transversely and stained with anti-zpr 1/FRet 43 (red) and either anti-complexin 4a (A, B, E, F) or anti-complexin 3/4 (C, D, G, H) (green). The complexin 4a translation-blocking morpholino dramatically decreases complexin 4a expression in the pineal organ (B) and in the retinal outer plexiform layer (OPL) and inner plexiform layer (IPL) (F). Some complexin 3/4 immunoreactivity remains in the pineal (D) and retina (H). Scale bars: 15 μm (A-D); 30 μm (E-H).

To confirm the specificity of this antibody for complexin 4a in zebrafish, a morpholino antisense oligonucleotide directed against the translation start site of complexin 4a was injected into embryos (Figure 11). Complexin 4a immunoreactivity is absent from the pineal organ of complexin 4a morphants at 1.5 dpf (data not shown). At approximately 4.3 dpf, complexin 4a immunoreactivity in the complexin 4a morphant pineal organ is still dramatically reduced (Figure 11B) compared to controls (Figure 11A). Other members of the complexin 3/4 subfamily are expressed in morphant pineal (Figure 11D), although there appears to be less expression of complexin 3/4 compared to control pineal organ (Figure 11C). Complexin 4a expression is also robustly decreased in both the OPL and IPL of the complexin 4a morphant retina at 4.3 dpf (Figure 11F). Complexin 3/4 immunoreactivity is most apparent in the complexin 4a morphant IPL, especially in large terminals in sublamina b (Figure 11H). Taken together, these results suggest that complexin 4a is a major complexin 3/4 isoform in visual system, but not acousticolateral system, ribbon terminals.

## Discussion

In this study, we isolated five zebrafish orthologs in the complexin 3/4 subfamily with several evolutionarily conserved features, and examined their expression with previously generated polyclonal antibodies [23]. Characterization of complexin 3/4 expression in the developing zebrafish nervous system reveals that members of this subfamily are enriched in ribbon-containing sensory neurons. In zebrafish, as in rodents, complexin 3/4 localizes to retinal ribbon presynaptic terminals. We confirm that zebrafish hair cells lack complexin 3/4 in their basolateral domains, and implicate complexin 3/4 as a component of their apical stereocilia. Taken together, these findings suggest that members of the complexin 3/4 subfamily may have multiple roles in ribbon-containing sensory neurons as discussed below.

### Characterization of zebrafish orthologs defines the complexin 3/4 subfamily in vertebrates

Many mammalian genes have two zebrafish orthologs due to an apparent genome duplication event in ray-finned fish [47,48]. Phylogenetic analysis of the complexin 3/4 subfamily reveals the presence of two complexin 3 orthologs and three complexin 4 orthologs in zebrafish. While zebrafish complexins 3a and 3b share 85% amino acid identity, zebrafish complexins 4a and 4b share 76% amino acid identity. Since the fifth zebrafish ortholog has higher amino acid identity with zebrafish complexin 4 paralogs (57 to 59%) compared with zebrafish complexin 3 paralogs (50 to 53%), we have named this divergent isoform complexin 4c. Potential medaka (ENSORLP00000022544) and *Xenopus *(ENSXETP00000014005) orthologs with high homology to zebrafish complexin 4c are located in the ENSEMBL database, which provides evidence for several *cplx *gene duplication events in the evolutionary history of teleosts and amphibians.

Vertebrate complexins 3 and 4 exhibit several conserved proteomic and genomic features that differentiate this subfamily from complexins 1 and 2. For example, a motif that defines vertebrate complexins 3 and 4 is the carboxy-terminal CAAX moiety (where C = cysteine, A = aliphatic, X = serine, threonine, or cysteine), which can undergo post-translational modifications that lead to the addition of a prenyl anchor and reversible attachment to membranes [23]. Targeting to membranes has important functional consequences for complexin 4 [23], and may also be accomplished through myristoylation since nearly all vertebrate complexins 3 and 4 (but not vertebrate complexins 1 and 2) have multiple myristoylation motifs (data not shown).

All vertebrate full-length members of the complexin 3/4 subfamily are encoded by three exons, including an invariant exon containing 88 nucleotides that encodes part of the putative core alpha helix that binds to an assembled SNARE complex [23]. Highest amino acid homology between vertebrate complexins occurs within this core alpha helix. Complexin 1 binding is thought to stabilize the SNARE complex [49] in a fusion-ready state, preventing completion of fusion via an accessory alpha-helix [50-52] until calcium enters the presynaptic terminal and binds to synaptotagmin 1 [53-55]. Complexin 3/4 subfamily members share substantial amino acid identity in the accessory alpha-helix (56 to 100% when comparing mouse, human and zebrafish orthologs), but this sequence is not conserved with complexin 1/2 subfamily members (approximately 25%). The amino terminus of complexin 1 has been shown to be necessary for exocytosis [50], but this region also diverges with complexin 3/4 subfamily members (approximately 25% amino acid identity). The functional consequences of these differences remain to be determined.

Complexin 3/4 double knockout mice exhibit reduced b-wave amplitudes and increased implicit times on electroretinograms. Disorganized cytomatrix components and floating ribbons in photoreceptor terminals suggest a presynaptic etiology for the defect in synaptic transmission in these mice [28]. Complexins 3 and 4 can rescue the defect in fast, calcium-triggered transmitter release in hippocampal neurons from complexin 1/2 double knockout mice [23]. However, the precise steps regulated by complexins 3 and 4 in neurotransmitter release at ribbon synapses remain unclear.

### Targeting of complexin 3/4 to different compartments of ribbon-containing sensory neurons

Few proteins involved in synaptic vesicle exocytosis are known to be selectively expressed in ribbon presynaptic terminals (reviewed in [5,6]). Therefore, the identification of ribbon-specific isoforms of these synaptic regulators is of special interest. We have extended a previous study describing expression of complexin 3/4 in retinal ribbon terminals [23] by showing that members of this subfamily are also enriched in photosensitive ribbon terminals of the zebrafish pineal organ. Knockdown of complexin 4a reveals that this isoform predominates in ribbon terminals of the zebrafish pineal and retina. Taken together, these results indicate that complexin 3/4 isoforms, such as complexin 4a in zebrafish, are markers for several types of visual system ribbon terminals.

Furthermore, we show that complexin 3/4 and another member of the exocytotic machinery, RIBEYE b, appear to be targeted concomitantly to photoreceptor terminals during zebrafish embryonic development. Since RIBEYE b is not expressed in bipolar cells [7], we were unable to analyze the time course of complexin 3/4 accumulation in bipolar cell terminals relative to a ribbon marker. However, it was observed that complexin 3/4 appeared to be targeted directly to bipolar cell terminals very rapidly after its clustering in retinal photoreceptor terminals (data not shown). Live imaging of fluorescently tagged components of the exocytotic machinery, expressed under the control of retinal bipolar cell [56,57] or other ribbon-containing neuron promoters [58], may shed additional light on the mechanisms and signals that regulate their targeting.

While complexin 3/4 localizes to the axons and presynaptic terminals of ribbon-containing sensory neurons in the zebrafish visual system, members of this subfamily are absent from the corresponding basolateral domains of hair cells in the zebrafish auditory, vestibular, and lateral line systems. Rather, complexin 3/4 immunoreactivity appears on the apical surfaces of these hair cells. The area around the hair cell cuticular plate contains large quantities of vesicles that traffic between the cytoplasm and plasma membrane [59]. Co-localization with the binding of phalloidin to F-actin above the cuticular plate suggests that complexin 3/4 is a component of stereocilia rather than vesicles near the apical plasma membrane. Immunoelectron microscopy will be necessary to determine the precise localization of zebrafish complexin 3/4 in the stereocilia.

During the course of this study, Strenzke *et al*. [60] utilized RT-PCR and immunocytochemistry with the two anti-complexin rabbit polyclonal antibodies to conclude that complexin 3/4 is not expressed in rodent inner ear hair cells. Our preliminary experiments confirm that complexin 3 is not expressed in hair cells of the adult mouse utricle (data not shown). Additional experiments are needed to examine the expression of complexin 3/4 during rodent hair cell development. In addition, since we focused in the embryonic zebrafish on the targeting of complexin 3/4 immunoreactivity in macular and otic neuromast hair cells, it will be useful to determine the spatiotemporal distribution profiles of complexin 3/4 in other hair cells of the inner ear and lateral line during embryonic development.

## Conclusions

Sensory receptor cells of the visual and acousticolateral systems are polarized into anatomically and physiologically distinct apical and basolateral compartments. Here we show that, in developing zebrafish, members of the complexin 3/4 subfamily are enriched and rapidly targeted to distinct compartments in these cells in a system-specific manner. These findings suggest that the functional and structural organization of ribbon-containing sensory receptor cells results from multiple direct targeting mechanisms during development.

## Materials and methods

### Zebrafish husbandry and tissue processing

All procedures were approved by the Institutional Animal Care and Use Committee at the State University of New York at Stony Brook. Wild-type zebrafish (*Danio rerio*) obtained from a local pet store [61] were propagated by natural matings and maintained on a controlled cycle of 13 hours of light and 11 hours of darkness at 28.5°C in system water [62]. Onset of fertilization was approximately 9:30 AM (30 minutes after lights were turned on). Embryos and larvae were staged as previously described [63].

Before overnight fixation at 4°C with 4% paraformaldehyde (Electron Microscopy Sciences, Hatfield, PA, USA), zebrafish embryos and larvae were anesthetized with 0.168 mg/ml tricaine (Sigma-Aldrich, St. Louis, MO, USA). For immunocytochemistry, fixed embryos and larvae were washed extensively in phosphate-buffered saline (PBS), cryoprotected in 30% sucrose until equilibrated, embedded in M-1 matrix (Shandon Lipshaw, Pittsburgh, PA, USA) and frozen at -80°C. Tissue was sectioned at 20 to 30 μm with a Leica CM1850 cryostat (Leica Microsystems, Bannockburn, IL, USA) with a chamber temperature of -20°C to -22°C and then stored at either -20°C or -80°C.

For isolation of zebrafish retinal RNA from freshly dissected eyes, adult zebrafish were decapitated, enucleated, and the lens and vitreous were extracted in cold, oxygenated, low-calcium solution containing (in mM): NaCl (102), KCl (2.5), MgCl_2 _(1), CaCl_2 _(0.5), glucose (10), and HEPES (10), pH 7.4. The retinae were removed from the eyecups, separated from retinal pigment epithelium, and incubated for 25 minutes in 500 units/ml hyaluronidase (Worthington Biochemical Corp., Lakewood, NJ, USA) at room temperature to remove residual vitreous. Retinae were rapidly frozen on dry ice, and poly(A) + RNA was obtained with the Micro Poly A + Pure kit (Applied Biosystems, Foster City, CA, USA).

### Mouse husbandry and tissue processing

Adult C57BL/6 mice (Taconic, Hudson, NY, USA) were euthanized by CO_2 _inhalation, decapitated, and brains were removed for access to the inner ears. The utricles were isolated in PBS, fixed for 2 hours in 4% paraformaldehyde at 4°C, washed extensively in PBS, and cryoprotected in 30% sucrose overnight. Adult mice were also enucleated, retinae were removed from the eyecups, separated from retinal pigment epithelium, and either placed in cold SDS sample buffer or fixed in 4% paraformaldehyde overnight at 4°C. Retinae were cryoprotected in 30% sucrose overnight, embedded in M-1 matrix and sectioned as described for zebrafish.

### Cloning and sequence analyses

The nucleotide sequences of mouse complexin 3 [GenBank:AY264290], human complexin 3 [GenBank:AY286501], mouse complexin 4 [GenBank:AY264291], and human complexin 4 [GenBank:AY286502] [23] were used in BLAST searches of the zebrafish genome in the ENSEMBL and GenBank databases. Five putative orthologs were identified in the zebrafish ENSEMBL database (Zv7) - ENSDARG00000062508, ENSDARG00000067826, ENSDARG00000059978, ENSDARG00000059486, and ENSDARG00000069963. To determine whether these genes are expressed in zebrafish, the following primers (which were expected to span multiple introns) were designed: (ENSDARG00000062508 forward) 5'-CGCGACTAACGTTAGGAATT-3'; (ENSDARG00000062508 reverse) 5'-AAAATCACCTCTGATCCTTG-3'; (ENSDARG00000067826 forward) 5'-GAGGAGATGAATCTACATCAC-3'; (ENSDARG00000067826 reverse) 5'-CTCCTAGAACAATATTAGCATC-3'; (ENSDARG00000059978 forward) 5'-ATGGCGTTTTTAATCAAAAGTATGG-3'; (ENSDARG00000059978 reverse) 5'-CTACATGACAGAACATTTCTCCTCG-3'; (ENSDARG00000059486 forward) 5'-GCTTCACATTCATTGTGATCAGCC-3' (ENSDARG00000059486 reverse) 5'-GGTCTCGAGGGATTGGAATGAC-3' (ENSDARG00000069963 forward) 5'-CAGTGTTACCTGTGCAGCTC-3'; (ENSDARG00000069963 reverse) 5'-CTCTCACATTAGCTGCACTCC-3'.

Reverse transcription of adult zebrafish retina poly(A) + RNA was carried out with Superscript II reverse transcriptase (Invitrogen, Carlsbad, CA, USA). PCR was performed with Platinum *Taq *DNA polymerase (Invitrogen), 1 μl of reverse-transcribed cDNA (or non-reverse transcription control), and 10 pmol of primers. The PCR protocol consisted of 95°C for 5 minutes, followed by 30 cycles of 95°C, 45°C, and 72°C for 1 minute each, ending with 72°C for 4 minutes. PCR products of approximate expected sizes, obtained with all five primer pairs, were gel-purified, subcloned in pGEM-T Easy, and sequenced. Comparison of these cDNA clones with the aforementioned annotated genes in the zebrafish ENSEMBL database via BLAST revealed >95% nucleotide identity for four of the genes (ENSDARG00000067826 was the exception).

Alignment of the cDNA clones and the aforementioned mammalian complexins 3 and 4 was performed with ClustalW in Molecular Biology Workbench [64]. Based on this multiple alignment, an unrooted phylogenetic tree was generated via the neighbor joining method and visualized with TreeView [65]. Bootstrap values supporting each node were calculated from 2,000 trials. Based on phylogenetic analysis, the cDNA clone most similar to ENSDARG00000062508 was named *cplx 3a*, ENSDARG00000067826 was named *cplx 3b*, ENSDARG00000059978 was named *cplx 4a*, ENSDARG00000059486 was named *cplx 4b*, and ENSDARG00000069963 was named *cplx 4c*. The sequences of the isolated cDNA clones have been deposited in GenBank: [GenBank:GU174497, GU174498, GU174499, GU174500, and GU174501] for zebrafish complexins 3a, 3b, 4a, 4b, and 4c, respectively.

*In silico *analysis of the following genes was performed based on sequence in the ENSEMBL database (Zv8): human *cplx 3 *(ENSG00000213578), human *cplx 4 *(ENSG00000166569), mouse *cplx 3 *(ENSMUSG00000039714), mouse *cplx 4 *(ENSMUSG00000024519), zebrafish *cplx 3a *(ENSDARG00000062508), and zebrafish *cplx 4a *(ENSDARG00000059978). Proteomic analysis of the cplx3/4 subfamily was performed in Molecular Biology Workbench with the PROSEARCH and PFSCAN algorithms.

### Construction and expression of myc-tagged complexins

Complexins 3a, 3b, 4a, 4b, and 4c were each fused in-frame with the myc tag from pCMV-Tag3b using the In-Fusion Dry-Down PCR Cloning Kit (Clontech Labs, Mountain View, CA, USA) according to the manufacturer's instructions. We used 50 pmol of the following primers to amplify 100 ng full-length complexins for eventual fusion with linearized pCMV-Tag3b: (complexin 3a forward) 5'-GAGCCCGGGCGGATCCATGGCTTTTATGTTGAAACACATG-3'; (complexin 3a reverse) 5'-GCAGCCCGGGGGATCCCTACATGACATCACACTTCTCGGC-3'; (complexin 3b forward) 5'-GAGCCCGGGCGGATCCATGGCTTTTATGGTGAAACACGTA-3'; (complexin 3b reverse) 5'-GCAGCCCGGGGGATCCTCACATGACGCAGCACTTCTCA-3'; (complexin 4a forward) 5'-GAGCCCGGGCGGATCCATGGCGTTTTTAATCAAAAGTATG-3'; (complexin 4a reverse) 5'-GCAGCCCGGGGGATCCCTACATGACAGAACATTTCTCCTC-3'; (complexin 4b forward) 5'-GAGCCCGGGCGGATCCATGTCTCATGATGGGATGTCC-3'; (complexin 4b reverse) 5'-GCAGCCCGGGGGATCCTCACATGACGGTGCATTTCTC-3'; (complexin 4c forward) 5'-GAGCCCGGGCGGATCCATGGCGTTCCTTCTGCAGCAG-3'; (complexin 4c reverse) 5'-GCAGCCCGGGGGATCCTCACATCAAGACACATTTTTCTTCC-3'.

HEK 293T cells were grown in Dulbecco's Modified Eagle's Medium (DMEM, Invitrogen) supplemented with 10% fetal bovine serum (HyClone Labs, Logan, UT, USA) and glutamine (Invitrogen) in a humidified incubator containing 5% CO_2 _and 95% air at 37°C. Cells on 100 mm dishes were transfected with Lipofectamine (Invitrogen) and 8 μg of a complexin-myc construct or pCMV-Tag3b. After 24 hours, the cells were trypsinized, plated onto poly-L-lysine-coated glass coverslips, and maintained for an additional 24 hours before processing for immunocytochemistry.

### Immunocytochemistry

HEK 293T cells were washed with PBS for 5 minutes, fixed with 4% paraformaldehyde for 15 minutes at room temperature, washed with PBS, permeabilized and blocked with PBS plus 0.3% Triton X-100 plus 6% normal goat serum (Jackson ImmunoResearch, West Grove, PA, USA) at room temperature for 2 hours. The coverslips were then incubated overnight at room temperature with mouse monoclonal anti-myc (Zymed Laboratories, San Francisco, CA, USA) and either rabbit polyclonal anti-complexin 3 or rabbit polyclonal anti-complexin 4 (Synaptic Systems, Göttingen, Germany) diluted in the blocking solution. In parallel, coverslips from each cell line were incubated solely with anti-myc, anti-complexin 3, anti-complexin 4, or the blocking solution. All of the coverslips were then washed three times with blocking solution and incubated for 1 hour at room temperature with affinity-purified, Alexa Fluor 488-conjugated goat anti-rabbit IgG and Alexa Fluor 546-conjugated goat anti-mouse IgG (Jackson ImmunoResearch) diluted 1:200 in the blocking solution. Finally, the coverslips were washed three times in PBS, once in distilled water, and mounted in Vectashield (Vector Laboratories, Burlingame, CA, USA).

Sections and wholemounts were washed with PBS and blocked for 2 hours at room temperature as described for the HEK293T cells. Primary antibodies (Table 1) were added to fresh blocking solution, and slides were incubated overnight at room temperature. For labeling of actin, Alexa Fluor 488 phalloidin (Invitrogen) was diluted 1:50 and incubated overnight with primary antibodies. After three washes, species-specific secondary antibodies were added for 1 hour at room temperature. Sections and wholemounts were washed three times with PBS, rinsed with water, and coverslipped in Vectashield.

**Table 1:** Primary antibodies used in this study

Antibody	Type	Dilution	Clone	Source	Immunogen	Characterization of specificity (reference)
Complexin 3	Rb pc	1:10,000		Synaptic Systems cat#122302	Full-length mo complexin 3	Immunofluorescence of heterologous cells expressing complexin isoforms; western blot (this study)
Complexin 4	Rb pc	1:75,000		Synaptic Systems cat#122402	Full-length mo complexin 4	Immunofluorescence of heterologous cells expressing complexin isoforms; immunofluorescence of morphant visual system (this study)
zpr 1/FRet 43	Mo mc	1:400		ZIRC	Adult zf retinal cells	Immunofluorescence of zf retina [31]
zn 1	Mo mc	1:40		ZIRC	Homogenized 1- to 5-dpf whole zf or membrane or basal lamina fractions	Immunofluorescence of zf inner ear [41]
PKC	Mo mc	1:1,000	MC5	BD Biosciences Pharmingen cat#554207	Bovine full-length PKC	Immunofluorescence of gf retina [32]
myc	Mo mc	1:250	9E10	Zymed Laboratories cat#13-2500	Hu c-myc amino acids 408-439	Antibody reacts specifically to hu c-myc amino acids 410-419 (manufacturer)
RIBEYE b	Rb pc	1:500		Dr T Nicolson	Zf RIBEYE b amino acids 133-483	Immunofluorescence of zf hair cells [36]
CtBP 2	Mo mc	1:1,000	16/CtBP 2	BD Biosciences Pharmingen cat#612044	Mo CtBP 2 amino acids 361-445	Western blot with retinal homogenates reveals expected bands of 120 and 110 kDa [15]

Cat#, catalog number; CtBP 2, carboxy-terminal binding protein 2; gf, goldfish; hu, human; mc, monoclonal; mo, mouse; pc, polyclonal; PKC, protein kinase C; rb, rabbit; zf, zebrafish; ZIRC, Zebrafish International Resource Center.

For labeling of neuromasts, live zebrafish larvae were bathed in 3.0 μM AM1-43 in embryo medium for 30 to 45 seconds [66]. After several washes in embryo medium. larvae were anesthetized with tricaine, fixed with 4% paraformaldehyde for 2 hours at room temperature, and cryoprotected in 30% sucrose overnight as described earlier. Larvae were sectioned sagittally at 30 μm, dried at room temperature, washed with PBS, and permeabilized with 0.3% Triton X-100 for 1 hour 15 minutes. Sections were then blocked for 2 hours with PBS plus 6% normal goat serum, and incubated overnight with primary antibodies in the blocking solution. Slides were processed the next day as described above, except for the use of affinity-purified, Alexa Fluor 546-conjugated goat anti-rabbit IgG (Jackson ImmunoResearch), to detect complexins.

### Data acquisition and image analysis

Images were acquired with either a FluoView 300 laser scanning confocal microscope running Olympus FluoView 5.0 or an Olympus FluoView FV1000 laser scanning confocal microscope running Olympus10-ASW software (Olympus America, Center Valley, PA, USA). For the Olympus FluoView FV300 confocal, images were collected with an Olympus UPlan-Apochromat 60 × NA 1.20 objective or a Zeiss Fluar 10 × NA 0.5 objective. For the Olympus FluoView FV1000 confocal, images were collected with an Olympus Plan-Apochromat 60 × NA 1.42 objective or an Olympus UPlan-S Apochromat 10 × NA 0.4 objective.

For all double-labeling experiments, sequential scans were taken to reduce crosstalk. For most experiments, a series of confocal optical sections (with a step size of 0.5 to 2.0 μm) was taken through the entire tissue section and a planar projection was generated. Images were passed through a median filter, and exported to ImageJ64 (NIH, Bethesda, MD, USA) and Photoshop CS3 (Adobe Systems Inc., San Jose, CA, USA), where levels were increased to reflect the original captures in FluoView.

To quantify the zebrafish complexin 3/4 reactivity profiles of the rabbit polyclonal anti-complexin 3 and anti-complexin 4 antibodies, 50 random fields of transfected HEK 293T cells were photographed from a representative of triplicate experiments for each cell line. All images were taken within the dynamic range of the photomultiplier, and identical gain and offset settings were used when imaging complexin (and myc) antibody immunoreactivity between cell lines. For each field, confocal z-stacks were taken through the entire cell with a step size of 2 μm, and the middle three optical sections were chosen to make a z-projection for each cell. These projections were encircled in ImageJ, and the mean pixel intensities were measured for both channels. Background fluorescence, which was obtained by measuring the mean pixel intensity for both channels in a region outside of a cell, was subtracted from the pixel intensity measurements. To compare the immunoreactivity of a complexin antibody directly, we calculated mean fluorescence ratios for each complexin paralog by dividing the corrected mean complexin antibody fluorescence intensity by the corrected mean myc fluorescence intensity. Student's *t*-tests were performed with Microsoft Excel 2008, with *P *< 0.05 considered to be statistically significant.

### Western blots

Total protein extracts were prepared from freshly isolated adult mouse and zebrafish retinae. Zebrafish extract was made in hypotonic lysis buffer (with protease inhibitors) and loaded at 200 μg, while mouse extract was loaded at 100 μg in SDS-sample buffer (with protease inhibitors). Proteins were size-fractionated by 15% SDS-PAGE and transferred to nitrocellulose membranes. The blot was probed with rabbit polyclonal anti-complexin 3 antibody (1:1,000) in tris-buffered saline tween-20 (TBST) plus 2.5% milk overnight, followed by DyLight 800 conjugated anti-rabbit IgG (Rockland Immunochemicals, Gilbertsville, PA, USA). Fluorescence was detected with the Odyssey Imaging System (LI-COR Biosciences, Lincoln, NE, USA).

### Morpholinos

To knock down complexin 4a protein expression, 5 ng of an antisense morpholino oligonucleotide complementary to the translation start site of the complexin 4a gene (5'-AAACGCCATTATTTACCACGCCGGA-3'; Gene Tools, Philomath, OR, USA) was pressure-injected in a volume of 0.5 nl into one-cell-stage, dechorionated zebrafish embryos. A control morpholino oligonucleotide (5 ng/0.5 nl) directed against human beta-globin (Gene Tools) was used as a negative control. Morpholino solutions were diluted to a final concentration of 10 ng/nl with 0.2 M KCl and 2 mg/ml phenol red. An additional negative control condition consisted of dechorionated zebrafish embryos that were not injected. All zebrafish embryos were then raised at 25.5°C in embryo medium (0.346 mg/ml sodium bicarbonate supplemented with Hanks Solution #1, Hanks Solution #2, Hanks Solution #4, Hanks Solution #5, and penicillin-streptomycin (Invitrogen)).

## Abbreviations

cplx: complexin; CSK: c-src kinase; dpf: days post-fertilization; ERGIC-53: ER Golgi intermediate compartment 53-kDa; hpf: hours post-fertilization; IPL: inner plexiform layer; lman1: lectin mannose-binding 1; OPL: outer plexiform layer; PBS: phosphate-buffered saline; RAX: retina and anterior neural fold homeobox gene.

## Competing interests

The authors declare that they have no competing interests.

## Authors' contributions

GZ conceived the study, carried out the experiments, prepared the figures, and wrote the initial manuscript. GM also conceived the study, revised the manuscript, and supervised the work, which was done in his laboratory.

## Supplementary Material

Additional file 1 **Identification of a pan-immunoreactive polyclonal antibody that recognizes zebrafish complexins 3 and 4**. **(A-J) **HEK 293T cells were transfected with myc-tagged, full-length zebrafish complexin 3a (A, B), 3b (C, D), 4a (E, F), 4b (G, H), or 4c (I, J). After 48 hours, cells were fixed with 4% paraformaldehyde and double-stained with a rabbit polyclonal antibody directed against mammalian complexin 3 (Synaptic Systems antibody 122302) (A, C, E, G, I) and a mouse monoclonal anti-myc antibody (B, D, F, H, J). This experiment was done in triplicate with a complexin antibody dilution of 1:10,000, and 50 random cells from one experiment were randomly selected for quantification. The mean green fluorescence intensity for each cell was divided by the mean red fluorescence intensity. **(K) **The mean fluorescence ratios and SEM for each cell line are shown, revealing that this antibody recognizes all five isoforms. Scale bar = 25 μm. Click here for file

Additional file 2 **The complexin 3/4 antibody predominantly recognizes an approximately 20 kDa band on western blots**. Adult mouse and zebrafish retinae were lysed in sample buffer containing protease inhibitors. Mouse extract (100 μg) and zebrafish extract (200 μg) were fractionated by SDS-PAGE (15% gel), blotted onto nitrocellulose, and probed with the complexin 3/4 polyclonal antibody (1:1,000). An approximately 20 kDa band is apparent in both mouse and zebrafish lysates. A minor band of approximately 17 kDa can also be observed in the zebrafish extract. Click here for file

Additional file 3 **Identification of a polyclonal antibody that preferentially recognizes zebrafish complexin 4a**. **(A-J) **HEK 293T cells were transfected with myc-tagged, full-length zebrafish complexin 3a (A, B), 3b (C, D), 4a (E, F), 4b (G, H), or 4c (I, J). These transiently transfected cells were stained with the mouse monoclonal anti-myc antibody (B, D, F, H, J) and a rabbit polyclonal antibody directed against mammalian complexin 4 (Synaptic Systems antibody 122402) (A, C, E, G, I). (E, F) Complexin and myc immunoreactivity, respectively, in a representative cell transfected with zf cplx4a-myc. This experiment was done in triplicate with a complexin antibody dilution of 1:10,000, and 50 random cells from one experiment were randomly selected for quantification. The mean green fluorescence intensity for each cell was divided by the mean red fluorescence intensity. **(K) **This antibody preferentially recognizes zebrafish complexin 4a (unpaired Student's *t*-test, *P *< 0.0001). Scale bar = 25 μm. Click here for file

Additional file 4 **Complementary expression of complexin 3 and complexin 4 in adult mouse retina**. Adult mouse retinal sections were labeled with anti-carboxy-terminal binding protein 2 (CtBP 2, red) and either anti-complexin 3 (A, green) or anti-complexin 4 (B, green). Since CtBP 2 and RIBEYE are transcribed from the same gene [67], anti-CtBP 2 can be used to label synaptic ribbons. A low-magnification confocal projection through a retinal section stained with anti-complexin 3 and anti-CtBP 2 (A) reveals complexin 3 expression in large, putative cone pedicles of the OPL and throughout most of the IPL, especially in the large, putative bipolar cell terminals in sublamina b. (B) Complexin 4 is found throughout the OPL in small terminals, which may be rod spherules, and in a thin band in the IPL that lacks complexin 3. Scale bar = 50 μm. Click here for file
