# Supplementary figures and images for: Enrichment and differential targeting of complexins 3 and 4 in ribbon-containing sensory neurons during zebrafish development
Source: Neural Dev. 2010 Sep 1;5:24. doi: 10.1186/1749-8104-5-24 (PMC2941751; doi:10.1186/1749-8104-5-24)

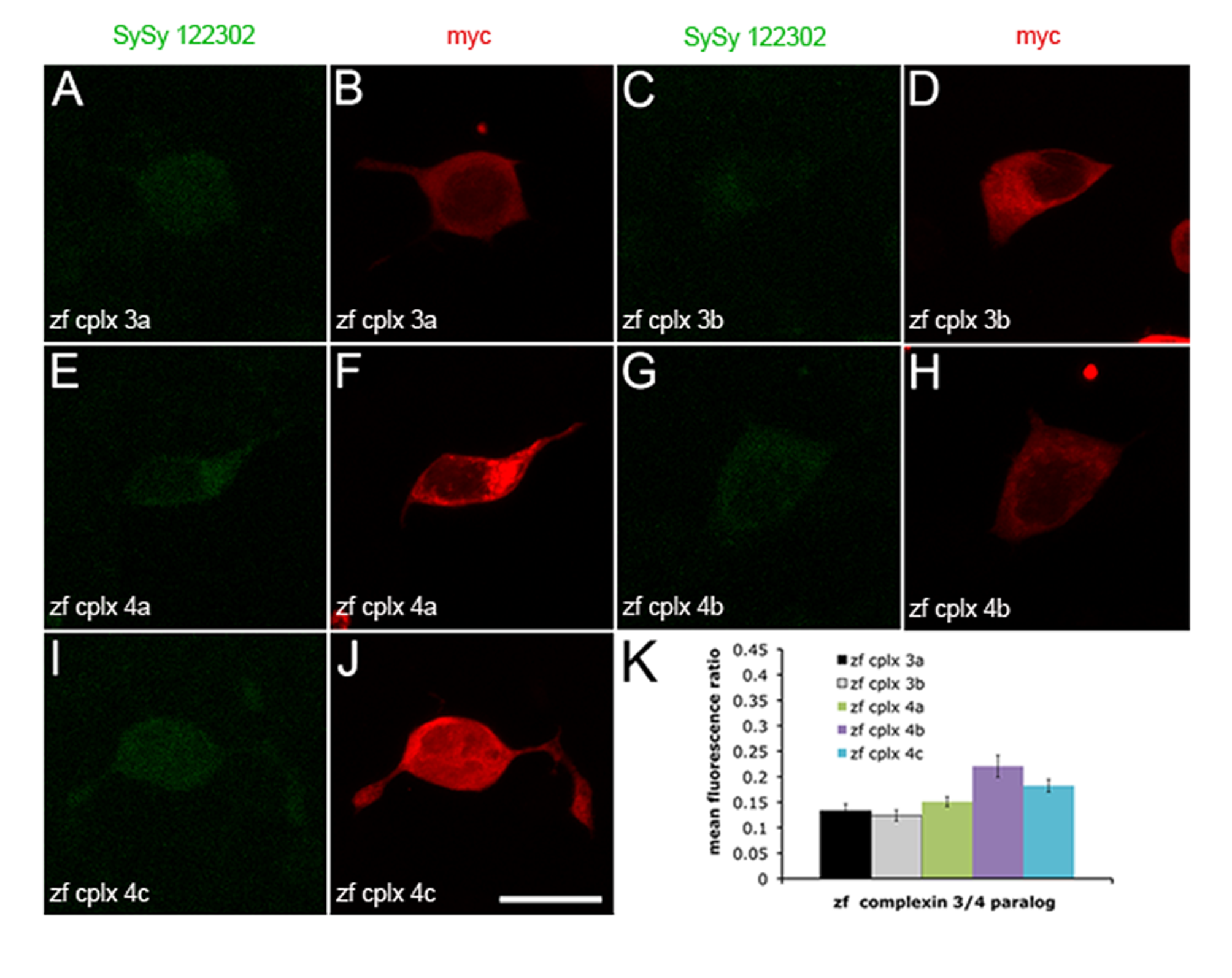

Supplement: Additional file 1 — Identification of a pan-immunoreactive polyclonal antibody that recognizes zebrafish complexins 3 and 4. (A-J) HEK 293T cells were transfected with myc-tagged, full-length zebrafish complexin 3a (A, B), 3b (C, D), 4a (E, F), 4b (G, H), or 4c (I, J). After 48 hours, cells were fixed with 4% paraformaldehyde and double-stained with a rabbit polyclonal antibody directed against mammalian complexin 3 (Synaptic Systems antibody 122302) (A, C, E, G, I) and a mouse monoclonal anti-myc antibody (B, D, F, H, J). This experiment was done in triplicate with a complexin antibody dilution of 1:10,000, and 50 random cells from one experiment were randomly selected for quantification. The mean green fluorescence intensity for each cell was divided by the mean red fluorescence intensity. (K) The mean fluorescence ratios and SEM for each cell line are shown, revealing that this antibody recognizes all five isoforms. Scale bar = 25 μm. [file 1749-8104-5-24-S1.TIFF]

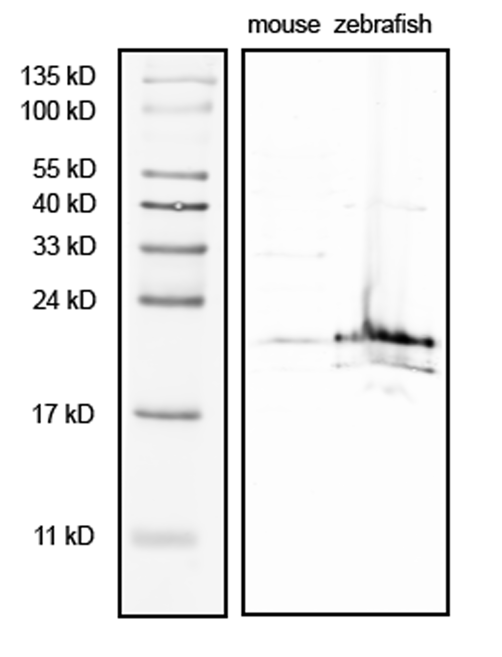

Supplement: Additional file 2 — The complexin 3/4 antibody predominantly recognizes an approximately 20 kDa band on western blots. Adult mouse and zebrafish retinae were lysed in sample buffer containing protease inhibitors. Mouse extract (100 μg) and zebrafish extract (200 μg) were fractionated by SDS-PAGE (15% gel), blotted onto nitrocellulose, and probed with the complexin 3/4 polyclonal antibody (1:1,000). An approximately 20 kDa band is apparent in both mouse and zebrafish lysates. A minor band of approximately 17 kDa can also be observed in the zebrafish extract. [file 1749-8104-5-24-S2.PNG]

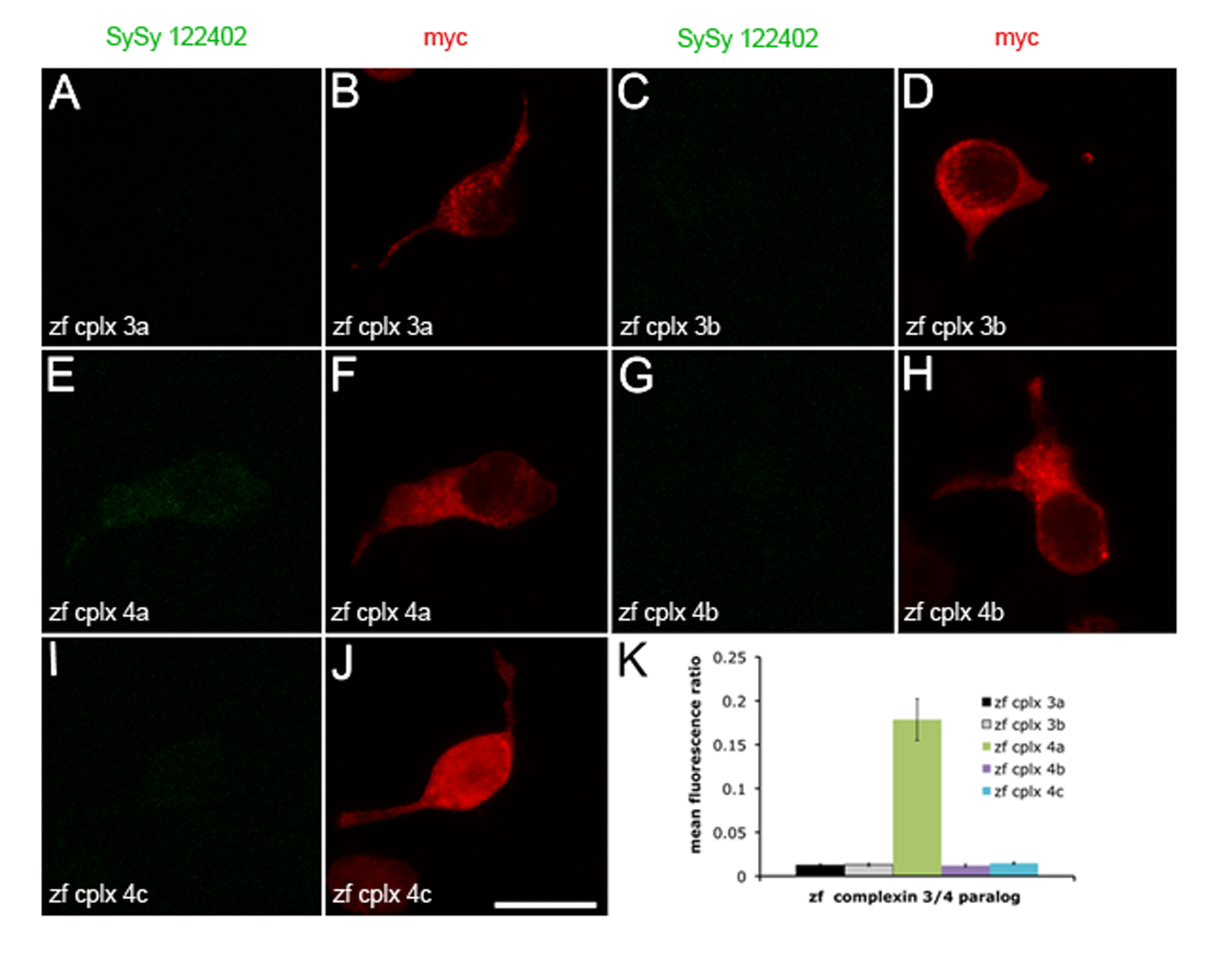

Supplement: Additional file 3 — Identification of a polyclonal antibody that preferentially recognizes zebrafish complexin 4a. (A-J) HEK 293T cells were transfected with myc-tagged, full-length zebrafish complexin 3a (A, B), 3b (C, D), 4a (E, F), 4b (G, H), or 4c (I, J). These transiently transfected cells were stained with the mouse monoclonal anti-myc antibody (B, D, F, H, J) and a rabbit polyclonal antibody directed against mammalian complexin 4 (Synaptic Systems antibody 122402) (A, C, E, G, I). (E, F) Complexin and myc immunoreactivity, respectively, in a representative cell transfected with zf cplx4a-myc. This experiment was done in triplicate with a complexin antibody dilution of 1:10,000, and 50 random cells from one experiment were randomly selected for quantification. The mean green fluorescence intensity for each cell was divided by the mean red fluorescence intensity. (K) This antibody preferentially recognizes zebrafish complexin 4a (unpaired Student's t-test, P < 0.0001). Scale bar = 25 μm. [file 1749-8104-5-24-S3.TIFF]

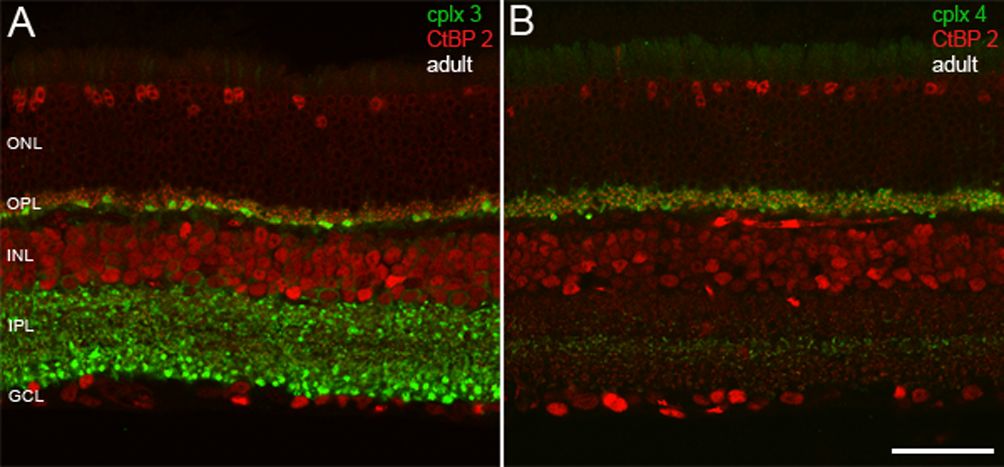

Supplement: Additional file 4 — Complementary expression of complexin 3 and complexin 4 in adult mouse retina. Adult mouse retinal sections were labeled with anti-carboxy-terminal binding protein 2 (CtBP 2, red) and either anti-complexin 3 (A, green) or anti-complexin 4 (B, green). Since CtBP 2 and RIBEYE are transcribed from the same gene [67], anti-CtBP 2 can be used to label synaptic ribbons. A low-magnification confocal projection through a retinal section stained with anti-complexin 3 and anti-CtBP 2 (A) reveals complexin 3 expression in large, putative cone pedicles of the OPL and throughout most of the IPL, especially in the large, putative bipolar cell terminals in sublamina b. (B) Complexin 4 is found throughout the OPL in small terminals, which may be rod spherules, and in a thin band in the IPL that lacks complexin 3. Scale bar = 50 μm. [file 1749-8104-5-24-S4.TIFF]
